# Endocrine Disruptors and Asthma-Associated Chemicals in Consumer Products

**DOI:** 10.1289/ehp.1104052

**Published:** 2012-03-08

**Authors:** Robin E. Dodson, Marcia Nishioka, Laurel J. Standley, Laura J. Perovich, Julia Green Brody, Ruthann A. Rudel

**Affiliations:** 1 Silent Spring Institute, Newton, Massachusetts, USA; 2 Battelle Memorial Institute, Columbus, Ohio, USA; 3 Clear Current LLC, Belmont, California, USA

**Keywords:** alkylphenols, asthma, bisphenol A, consumer products, cyclosiloxane, endocrine disruptors, fragrance compounds, parabens, phthalates, UV filters

## Abstract

Background: Laboratory and human studies raise concerns about endocrine disruption and asthma resulting from exposure to chemicals in consumer products. Limited labeling or testing information is available to evaluate products as exposure sources.

Objectives: We analytically quantified endocrine disruptors and asthma-related chemicals in a range of cosmetics, personal care products, cleaners, sunscreens, and vinyl products. We also evaluated whether product labels provide information that can be used to select products without these chemicals.

Methods: We selected 213 commercial products representing 50 product types. We tested 42 composited samples of high-market-share products, and we tested 43 alternative products identified using criteria expected to minimize target compounds. Analytes included parabens, phthalates, bisphenol A (BPA), triclosan, ethanolamines, alkylphenols, fragrances, glycol ethers, cyclosiloxanes, and ultraviolet (UV) filters.

Results: We detected 55 compounds, indicating a wide range of exposures from common products. Vinyl products contained > 10% bis(2-ethylhexyl) phthalate (DEHP) and could be an important source of DEHP in homes. In other products, the highest concentrations and numbers of detects were in the fragranced products (e.g., perfume, air fresheners, and dryer sheets) and in sunscreens. Some products that did not contain the well-known endocrine-disrupting phthalates contained other less-studied phthalates (dicyclohexyl phthalate, diisononyl phthalate, and di-*n*-propyl phthalate; also endocrine-disrupting compounds), suggesting a substitution. Many detected chemicals were not listed on product labels.

Conclusions: Common products contain complex mixtures of EDCs and asthma-related compounds. Toxicological studies of these mixtures are needed to understand their biological activity. Regarding epidemiology, our findings raise concern about potential confounding from co-occurring chemicals and misclassification due to variability in product composition. Consumers should be able to avoid some target chemicals—synthetic fragrances, BPA, and regulated active ingredients—using purchasing criteria. More complete product labeling would enable consumers to avoid the rest of the target chemicals.

Chemicals contained in consumer products are ubiquitous in human tissues, sometimes at high concentrations [Centers for Disease Control and Prevention (CDC) 2009] and in household air and dust (Rudel and Perovich 2009; Rudel et al. 2003, 2010; Weschler 2009). Studies of pesticides, polychlorinated biphenyls (PCBs), polybrominated diphenyl ether (PBDE) flame retardants, and volatile organic compounds (VOCs) in homes provide some information about sources, exposure pathways, and exposure reduction options (Dodson et al. 2008; Lorber 2008; Rudel et al. 2008; Zota et al. 2008). However, for many common commercial chemicals, limited information is available about how specific consumer products contribute to exposure. In particular, little information is available about exposures from personal care and cleaning products.

Many of these products may be sources of chemicals that have a diverse spectrum of health effects, including endocrine disruption and associations with asthma. Endocrine-disrupting compounds (EDCs) are chemicals that can alter hormonal signaling and have potential effects on developing reproductive and nervous systems, metabolism, and cancer (Colborn et al. 1993). Some phthalates inhibit testosterone synthesis (Howdeshell et al. 2008), and antimicrobials such as triclosan suppress thyroid hormone (Paul et al. 2010) and are estrogenic (Stoker et al. 2010) in mammalian models. Some parabens, alkylphenols, cyclosiloxanes, ultraviolet (UV) filters, and synthetic musk fragrance compounds are weakly estrogenic in a variety of experimental models (Bitsch et al. 2002; Bonefeld-Jørgensen et al. 2007; Quinn et al. 2007; Routledge et al. 1998; Schlumpf et al. 2004; Schreurs et al. 2005). Factors related to home environments are associated with asthma, although there has been limited study of the role of chemical contaminants (Douwes and Pearce 2002). Fragrances have been shown to exacerbate asthma (Kumar et al. 1995). The phthalate bis(2-ethylhexyl) phthalate (DEHP) in dust was associated with asthma and wheezing in children (Bornehag et al. 2004), and several phthalates show an adjuvant effect in animal studies (Bornehag and Nanberg 2010). The sum of propylene glycol and glycol ethers was associated with increased asthma prevalence in preschool-age children (Choi et al. 2010). The ethanolamines monoethanolamine and diethanolamine are occupational asthmagens (Association of Occupational and Environmental Clinics 2010).

Previous research suggests that consumer products are a source of these compounds in homes. We found a wide range of phthalates, alkylphenols, parabens, flame retardants, PCBs, and current-use and banned pesticides in air and dust samples from homes, with 13–28 compounds in air and 6–42 compounds in dust (Rudel et al. 2003). Analysis of paired indoor and outdoor air samples in California demonstrated that indoor concentrations were considerably higher than outdoor concentrations for many compounds, indicating the constant presence of indoor sources (Brody et al. 2009; Rudel et al. 2010).

Efforts to identify the contribution of specific products to home environments or personal exposure are hindered by limited and inconsistent disclosure of chemical ingredients in consumer products. Regulations require only limited labeling. For example, sunscreens, antiperspirant deodorants, and antibacterial hand soaps are regulated as over-the-counter drugs by the U.S. Food and Drug Administration (FDA), and “active” ingredients must be labeled (Fair Packaging and Labeling Act 1967; Federal Food, Drug, and Cosmetic Act 1938). For cosmetics, the FDA requires the listing of ingredients in order of predominance, except chemical constituents of fragrances and “incidental ingredients” do not need to be listed (Fair Packaging and Labeling Act 1967; Federal Food, Drug, and Cosmetic Act 1938). For cleaning products, ingredient labeling is required only for compounds, such as antimicrobials, that are regulated by U.S. Environmental Protection Agency (EPA) under the Federal Insecticide, Fungicide, and Rodenticide Act (FIFRA 1972). The labeling terms “natural,” “nontoxic,” and “green” are unregulated and require no standardized ingredient information. Indeed, in a recent study Steinemann et al. (2011) found that the VOC composition of “green”-labeled fragranced products was not significantly different from that of other fragranced products with regard to number of hazardous chemicals as defined under U.S. federal laws.

Gaps in ingredient information are problematic from multiple perspectives. Regulators rely on product ingredient concentrations for exposure modeling. Consumers want ingredient information so they can make precautionary choices consistent with personal values; although environmental health organizations have developed rating systems to advise consumers, these ratings are limited to information on product labels (Environmental Working Group 2011; GoodGuide 2012). In addition, researchers need ingredient information to interpret health studies and test exposure reduction strategies. In an effort to fill this gap, in 2007 we provided a list of EDCs to 34 manufacturers and asked them whether specific personal care and cleaning products contained those EDCs, but many were unwilling to provide the information (Dunagan et al. 2011).

To develop information about exposure sources, we characterized the concentrations of 66 chemicals in 50 types of household products, focusing on cleaners and personal care products. We also aimed to identify the predominant exposure sources in order to target for product substitution in an intervention study. Such intervention designs are powerful approaches to exposure assessment and have been used to estimate exposures to bisphenol A (BPA) and phthalates via food packaging (Rudel et al. 2011) and pesticide exposure from food (Lu et al. 2006). To identify substitute products for use in an intervention study, we tested samples of “alternative” products selected because their labels indicated that they might be free of the chemicals of concern. Thus, results also provide insight into the usefulness of product labeling for consumers seeking to reduce exposures.

## Methods

We selected 66 organic chemicals for inclusion in the study based on evidence of endocrine disruption or asthma exacerbation, expected presence in consumer products, and compatibility with analytical methods developed in our household exposure studies (Rudel et al. 2003, 2010). We tested 85 samples representing 213 products in two rounds of chemical analysis. The chemical groups, their typical uses, and the evidence of endocrine disruption or asthma exacerbation are listed in Supplemental Material, Table S1 (http://dx.doi.org/10.1289/ehp.1104052).

*Product selection.* We first identified the types of products likely to contain the compounds of interest. Product types included personal care products (e.g., lotion, hair products, toothpaste), cleaners (e.g., laundry detergent, all-purpose cleaner), and other household goods. We then identified several “conventional” products and one “alternative” for each product type. Exclusion criteria for alternative products are listed in Table 1. A product was classified as alternative if the label did not include the terms listed in Table 1. Many of the products that met our criteria for alternative products were marked as “green.” We also identified as alternative products six items often used in recipes for homemade cleaners, such as bleach and vinegar. Products that did not meet the “alternative” criteria were classified as conventional. In selecting conventional products, we tried to choose products that are widely used in order to better represent typical exposures. Because we lacked comprehensive information from which to select products, we identified leading companies for the product sector (e.g., hair care) based on market share and selected candidate products from several leading companies. When possible, we also included a generic store-brand product. Final product selections were made informally on the basis of availability and shelf space.

**Table 1:** Exclusion criteria for alternative products.

Term	Reason
Parabens		EDC (Kang et al. 2002)
Ethanolamines		Asthma-related (Kamijo et al. 2009; Mäkelä et al. 2011; Piipari et al. 1998; Savonius et al. 1994)
1,4-Dichlorobenzene		Carcinogen (IARC 1999)
Nonionic surfactants		Suggests alkylphenol-based ingredients, which are EDCs (Jie et al. 2010)
Fragrances other than “natural fragrances”a		Asthma-related (Kumar et al. 1995) and EDC (Bitsch et al. 2002; Seinen et al. 1999)
Tea tree oil, lavender		EDC (Henley et al. 2007)
Triclosan, triclocarban		EDC (Chen et al. 2008; Stoker et al. 2010)
Antibacterial		Suggests the presence of triclosan or triclocarban
Stain-resistant		Suggests organofluorines
Vinyl		Assumed to contain phthalates
Petroleum-based		Health concerns about petroleum derivatives
Products having these terms on the product label were excluded as alternative products and were thus considered conventional products. a“Natural fragrances” includes ingredients labeled as essential oils, plant-based fragrances, and other similar ingredients, which were allowed even though some individuals may be sensitive.		

We purchased most alternative products at a nationwide store specializing in natural products, so products met the store’s selection criteria, which favored non–petroleum-based—and especially plant-based—ingredients. Most of the conventional products were purchased at major grocery and pharmacy chain stores primarily in fall 2007. We added products for a second round of chemical analysis approximately 1 year later. Names of the products that were tested and their manufacturers are available from Silent Spring Institute (2012).

*Sampling design and compositing*. We analyzed 42 analytical samples composited from 170 conventional products and 43 samples of individual alternative products.

To cost-effectively evaluate typical exposures from conventional products, we composited 170 conventional products into a single sample for each product type (42 analytical samples). We combined equal masses of 1–7 products within a product type and analyzed the mixture as a single sample. The advantage of compositing is that samples may provide more generalizable exposure information. However, composited samples are more limited in that they *a*) will not reveal an unusually high concentration in a single product if that product is mixed with others having lower concentrations; *b*) will not reveal a concentration just above the limit of detection (LOD) in a single product if that product is mixed with others having concentrations < LOD; and *c*) may show a higher detection frequency for chemicals well within the detectable range.

We sought to identify specific products that were free of the chemicals of concern (alternative products), so the products could be used in an intervention study. Thus, we analyzed just 1 alternative product per product type (43 analytical samples, 1 for each of 43 individual products). Therefore, reported detection frequencies and concentrations for conventional and alternative product types are not directly comparable. To provide some information about variability in products within a category, we tested individual samples of 5 alternative sunscreens and calculated an average for the product type “alternative sunscreen.”

*Chemical analysis.* We analyzed samples in two rounds: 50 compounds in the first round and those same 50 compounds plus 16 other compounds in the second round. Products were composited as described above, and surrogate recovery standards were added. Samples were then extracted with dichloromethane:methanol, passed through a weak anion exchange cartridge, spiked with internal standard, and analyzed by gas chromatography/mass spectrometry in the full scan mode. A separate aliquot was derivitized and analyzed for phenolic compounds.

For each compound, the method reporting limit (MRL) was defined as the maximum analytical LOD and the 90th percentile of the blank concentrations within each analytical round. The reporting limit was 1 µg/g for chemicals in products, but it was reported as > 1 µg/g if there were detectable concentrations in the blank samples (1 chemical in analytical round 1 and 12 chemicals in analytical round 2).

We included extensive quality assurance/quality control (QA/QC) samples in our analyses. Chemical detection in blanks was infrequent, and elevated MRLs were ≤ 5 µg/g except for cyclosiloxane decamethylcyclopentasiloxane (D_5_; the only compound detected in > 75% of blanks). Results were blank corrected by subtracting the median blank value from the reported value. Precision was assessed with 13 duplicate samples (relative percent difference was generally < 50%); accuracy was assessed by determining spike recovery for all target compounds in six different matrices (median recoveries across products were generally within 50–150%) and by calculating recoveries of surrogates in all samples (median percent recoveries were within the 50–150% acceptance range for all surrogates in both analytical rounds). For additional details regarding chemical analysis and QA/QC measures, see Supplemental Material, pp. S-9–S-10 (http://dx.doi.org/10.1289/ehp.1104052).

**Figure 1:**
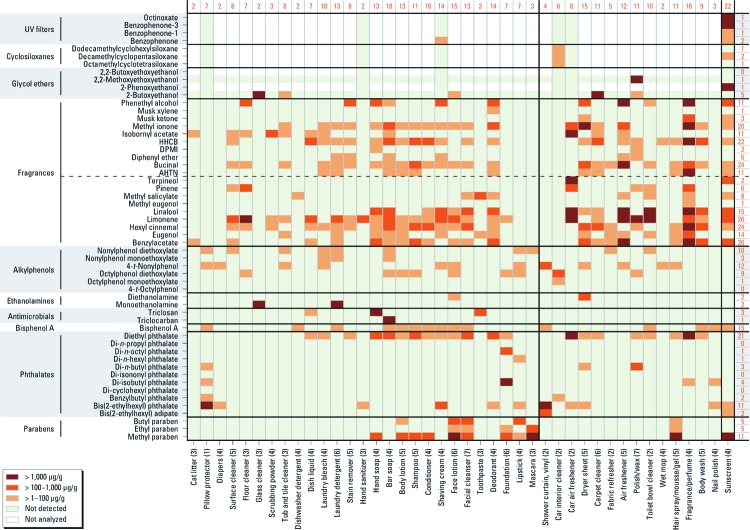
Concentrations of target compounds (left) in conventional consumer products (bottom) by product type. Compounds are grouped by chemical class, with natural and synthetic fragrances distinguished by a dashed horizontal line within the figure. Numbers in parentheses after product type indicate number of products in the composite. Numbers at the top of the figure indicate the number of chemicals detected in each product type; numbers on the right indicate the number of products containing each compound. The first 27 product types (left of the solid vertical line) and the last product type (sunscreen) are also shown in Figure 2, but the remaining product types differ.

**Figure 2:**
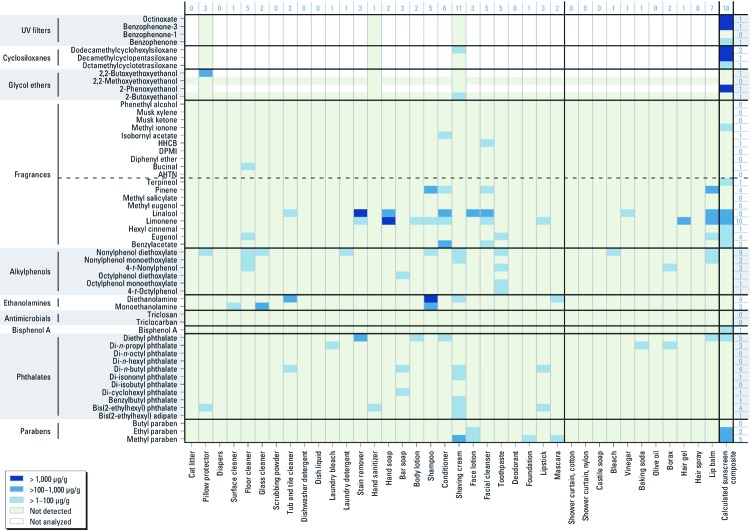
Concentrations of target compounds (left) in “alternative” consumer products (bottom) by product type. Compounds are grouped by chemical class, with natural and synthetic fragrances distinguished by a dashed horizontal line in the figure. Numbers at the top of the figure indicate the number of chemicals detected in each product type; numbers on the right indicate the number of products containing each compound. The first 27 product types (left of the solid vertical line) and the last product type (sunscreen) are also shown in Figure 1, but the remaining product types differ.

*Data analysis.* Our analysis of this large data set is visual and exploratory. We graphed product type against compounds detected using a “heat map” approach for conventional and alternative products (Figures 1 and 2, respectively). Only values > MRL or > 1 µg/g are presented. We graphed results for sunscreens in a similar format [see Supplemental Material, Figure S1 (http://dx.doi.org/10.1289/ehp.1104052); results are presented for a composited sample of conventional sunscreens, the calculated composite obtained by averaging results for five alternative sunscreens, and individual results for the five alternative sunscreens).

To identify chemicals that tend to co-occur because they are used together in a product, we estimated correlations for chemicals simultaneously detected within a product type (e.g., laundry detergent, lipstick). We calculated Kendall’s *tau* adjusted for censored data and with *p*-values obtained from 10,000 bootstrap replications (Newton and Rudel 2007). The magnitude of Kendall’s *tau* coefficients tends to be smaller than those of the more familiar Spearman’s correlation coefficients. We limited this analysis to chemicals detected in more than three analytical samples, and we conducted analyses separately for conventional and alternative products.

## Results and Discussion

We tested 213 conventional and alternative consumer products in 85 analytical samples for up to 66 compounds of interest. We detected 55 compounds: 50 chemicals in 42 conventional samples representing 170 products, and 41 compounds in 43 alternative samples representing 39 product types.

The most frequently detected compounds in conventional products were two natural fragrance compounds that may be derived from plant materials, two synthetic fragrance compounds, and diethyl phthalate (DEP) (Figure 1). The most frequently detected compounds in alternative products, including the calculated composite sunscreen, were two natural fragrance compounds, an alkylphenol, methyl paraben and DEP (Figure 2).

We detected 11 compounds at concentrations > 1% (10,000 µg/g) and 26 compounds at > 0.1%. DEHP was detected at 28% and 14% by weight in the vinyl shower curtain composite and the vinyl pillow protector, respectively. The glycol ether 2-butoxyethanol was detected at nearly 5% in the carpet cleaner. The sunscreen samples contained 2.5–6.2% of the UV filters octinoxate and benzophenone-3 (BP-3). The fragrance/​perfume composite contained almost 3% hexahydrohexamethyl cyclopentabenzopyran (HHCB), a synthetic fragrance chemical, and 1.4% DEP. The car air freshener contained the fragrance chemicals isobornyl acetate and limonene (a natural fragrance compound) at approximately 2% each. The alternative shampoo sample had 2.4% diethanolamine. Additional findings are described by chemical class.

*Parabens.* Parabens are added to many consumer products, pharmaceuticals, and foods as preservatives and antimicrobial agents (Soni et al. 2001). Previous studies found parabens, particularly methyl paraben, in most cosmetic samples (Rastogi et al. 1995; Shen et al. 2007). In a study of 100 demographically diverse adults, Ye et al. (2006) detected methyl and propyl paraben in > 96% of urine samples. Parabens are weakly estrogenic *in vitro*, and butyl paraben (100 mg/kg) has been reported to affect reproductive tract development in rats (Kang et al. 2002).

We detected parabens in personal care products but not in cleaners. Methyl paraben was detected most frequently and at the highest concentrations; ethyl and butyl paraben were found only if methyl paraben was also detected. The highest concentration was in an alternative sunscreen (methyl paraben; 1,600 µg/g). Of the 11 conventional samples with detectable parabens, 10 included products with “paraben” on the label. With the exception of shaving cream, products were not considered alternative if parabens were listed as an ingredient. Nevertheless, in alternative products, we detected parabens in 7 products, including 3 sunscreens, that did not list parabens on the label.

*Phthalates.* Phthalates are used as plastic additives, as solvents in cosmetics and perfumes, and as an inert ingredient in pesticides. Higher molecular weight phthalates (e.g., DEHP) are typically used in plastics (10–60% by weight) and readily migrate out of products (Rakkestad et al. 2007). Lower molecular weight phthalates [e.g., di-*n*-butyl phthalate (DBP), DEP] are typically used as solvents in personal care products and in lacquers, varnishings, and coatings (Meeker et al. 2009b). Several different phthalates have been reported in cosmetics and other personal care products, sometimes at concentrations > 1% (Hubinger and Havery 2006; Koniecki et al. 2011; Shen et al. 2007). Near universal detection of phthalates in urine samples shows widespread exposure (CDC 2009; Heudorf et al. 2007). In humans, phthalates have been associated with adverse reproductive system outcomes, including reduced semen quality and altered male genital development, as well as respiratory symptoms (Bornehag et al. 2004; Engel et al. 2010; Hauser and Calafat 2005; Hauser et al. 2006; Kimber and Dearmna 2010; Meeker et al. 2009a, 2009b; Mendiola et al. 2011; Swan 2008; Swan et al. 2005). Many phthalates are identified as antiandrogenic EDCs in mammalian models, whereas DEP is not generally characterized as an endocrine-active compound (Hannas et al. 2011; Heindel et al. 1989; Howdeshell et al. 2008). Among the EDCs in the present study, phthalates are the only chemical group for which there is supporting evidence of health effects from human studies.

We analyzed samples for 12 phthalates. DEP, a common solvent for fragrance (Hubinger and Havery 2006), was detected most frequently; the highest DEP concentrations were in fragrance/perfume (14,000 µg/g) and car air freshener (8,000 µg/g). Vinyl products had the highest concentrations of any phthalate, with DEHP at 28% in the shower curtains and 14% in the pillow protector. DBP and benzylbutyl phthalate (BBP) were detected in the conventional pillow protector, dryer sheet, polish/wax, car interior cleaner, and in alternative tub/tile cleaner, bar soap, shaving cream, and lipstick. Three phthalates were found only in alternative products: dicyclohexyl phthalate (DCP), diisononyl phthalate (DINP), and di-*n*-propyl phthalate (DPP). These compounds may have been introduced as substitutes for the better-known antiandrogenic phthalates (DBP, BBP, DEHP), even though they are also EDCs and have similar (DCP) or lesser (DINP, DPP) potency (Boberg et al. 2011; Hannas et al. 2011; Heindel et al. 1989; Saillenfait et al. 2009). The alternative shaving cream contained 5 different phthalates, illustrating the potential for simultaneous exposures to multiple phthalates, which act cumulatively on endocrine targets (National Research Council 2008). None of the products we tested had “phthalate” on the label, including personal care products requiring that intentional ingredients must be labeled. However, the conventional nail polish sample with measurable DEHP contained a product labeled “phthalic anhydride copolymer.”

*BPA.* BPA is used in a variety of consumer products containing epoxy resins, polyester-styrene, and polycarbonate plastics. It can be an ingredient in vinyl and in dental sealants, protective coatings, flame retardants, and adhesives (Meeker et al. 2009b). Biomonitoring studies indicate that exposure is widespread; BPA was detected in > 93% of urine samples in the National Health and Nutrition Examination Survey (NHANES) (Calafat et al. 2008b). A wide body of laboratory evidence shows BPA-induced endocrine disruption in a number of organ systems (Food and Agriculture Organization of the United Nations/World Health Organization 2010).

We detected BPA in 15 conventional samples, including the vinyl shower curtain and pillow protector, dish and laundry detergent, tub and tile cleaner, soaps, lotions, shampoo, conditioner, shaving cream, nail polish, and sunscreen. Concentrations were < 100 µg/g, with most < 10 µg/g. BPA was not detected in alternative samples except sunscreen, so selecting alternative products according to our criteria appears to avoid BPA. No labels listed BPA.

*Antimicrobials.* We analyzed four antimicrobials: *ortho*-phenylphenol, triclocarban, triclosan, and 1,4-dichlorobenzene. Both triclocarban and triclosan are broad-spectrum agents commonly added to personal care products, such as toothpastes and soaps, detergents, toys, plastics, and textiles (Adolfsson-Erici et al. 2002; Calafat et al. 2008c; Perencevich et al. 2001). A national survey detected triclosan and triclocarban in 76% of liquid soaps and 29% of bar soaps (Perencevich et al. 2001), and triclosan was detected in 76% of NHANES urine samples (Calafat et al. 2008c). Triclosan has been shown to disrupt thyroid homeostasis in mammalian models (Paul et al. 2010; Rodríguez and Sanchez 2010), and current human exposure levels are in the range of those predicted to have this activity based on laboratory tests (Rotroff et al. 2010). Triclocarban has been shown to amplify endogenous androgen response in mammalian models (Chen et al. 2008). Personal care product labels must list antimicrobial concentrations (FDA 2009).

We detected triclocarban and triclosan but not the other two antimicrobials. When they were detected at higher concentrations, they were listed as active ingredients on the product labels, consistent with FDA labeling requirements. For example, the conventional bar soap sample contained triclocarban at 1,520 µg/g, and one of the four soaps in the composite was labeled “antibacterial” with triclocarban (0.6%). Concentrations of triclosan in conventional hand soap and toothpaste were slightly lower than predicted from labeling of active ingredient concentrations. Some products (conventional facial cleanser and lipstick) listed triclosan in the ingredient list but not as an active ingredient; however, we did not detect it in those composite samples. Also, we found relatively low levels (6 µg/g) of triclosan in conventional dish liquid composite, although it was not listed on the product labels. We did not detect these compounds in any of the alternative products.

*Ethanolamines.* Monoethanolamine (MEA) is used in cleaners and degreasers, detergents, soaps, cosmetics, hair dyes, and as an emulsifier in lotions and creams; diethanolamine (DEA) is used as an emulsifier in shampoos, cleaners, detergents, polishes, and auto products (National Library of Medicine 2010b). Exposure studies are limited. MEA and DEA have been associated with occupational asthma (Association of Occupational and Environmental Clinics 2010; Kamijo et al. 2009; Mäkelä et al. 2011; Piipari et al. 1998; Savonius et al. 1994). The European Commission prohibits DEA in cosmetics and restricts products with MEA to < 0.5% amine content because of concerns about formation of carcinogenic nitrosamines (European Commission 2011).

We detected MEA in conventional glass cleaner and laundry detergent (> 1,000 µg/g) and in alternative surface cleaner, glass cleaner, and shampoo (< 400 µg/g). The highest DEA concentration was in the alternative shampoo (24,000 µg/g; 2.4% by weight). DEA was detected in the composite sample of conventional dryer sheets (840 µg/g) and in four other conventional and alternative cleaning and personal care product samples (< 150 µg/g): conventional face lotion and alternative tub and tile cleaner, mascara, and shaving cream.

No product labels listed MEA or DEA as ingredients. Several product labels included the derivatives “cocamide MEA,” “lauramide DEA,” or “cocamide DEA,” but MEA and DEA were not detected in samples of these products. We detected DEA in mascara labeled with triethanolamine (TEA), but MEA and DEA were not detected in other TEA-labeled products. Commercial mixtures of TEA may contain small amounts of DEA and MEA [International Agency for Research on Cancer (IARC) 2000].

*Alkylphenols.* Alkylphenol polyethoxylates (APEOs), including nonylphenol and octylphenol ethoxylates, are used as surfactants in consumer products such as detergents, disinfectants, and surface cleaners, and as “inert” ingredients in pesticides. Mixtures containing ethoxylate chains of varying lengths are typical and can degrade to nonylphenol and octylphenol, both identified as weakly estrogenic (Jie et al. 2010). Nonylphenol also originates from vinyl and other plastics containing tris(nonylphenol) phosphite and may have other uses, including in epoxy resins. The branched chain *para*-substituted nonylphenol and octylphenol are the commercially prevalent compounds (European Commission 2002; Rudel and Perovich 2009; Rudel et al. 2010).

We measured 4-*t*-octylphenol and 4-*t*-nonylphenol, as well as their mono and diethoxylates, and detected them in about half of the samples, including plastics, cleaners, and personal care products. Concentrations were low (< 20 µg/g) except in the conventional car cleaner and vinyl shower curtain.

Product labels did not list alkylphenols. Of the 37 alkylphenol-containing samples, 7 included products labeled as containing “surfactants” of any type (e.g., ionic, nonionic). Ten samples contained at least one product labeled as containing “surfactants,” but alkylphenols were not detected. Because many products containing APEOs did not list surfactants on labels, a significant fraction of the products may contain 4-*t*-nonylphenol from other uses, such as plasticizers, or are simply unlabeled. Thus, it appears that exposure to alkylphenols cannot be avoided by reading product labels.

*Fragrances.* Fragrances are added to products to achieve a desired scent or mask other scents in the product. More than 3,000 fragrance ingredients have been reported, and a fragranced product may contain 50–300 different chemicals (Bickers et al. 2003). Exact formulations are typically protected from disclosure (Bridges 2002; International Fragrance Association 2010). Fragrances can be either synthetic or natural/plant-based; however, some natural fragrance chemicals can be artificially synthesized, and these may or may not reflect the natural stereoisomer composition (Ravid et al. 2010) and may have different health effects (Smith 2009). In the present study we classified fragrance chemicals as natural if they are readily available from plant materials and synthetic if they are most commonly synthesized, based on information in the Hazardous Substances Data Bank (National Library of Medicine 2010a); however, we did not independently verify that the natural fragrance chemicals were not synthesized. Synthetic fragrance compounds, which include polycyclic and nitro musks, have been found in many personal care and household products and at concentrations up to several thousand micrograms per gram (Reiner and Kannan 2006; Zhang et al. 2008). Synthetic and natural fragrance compounds have been reported in cleaning products (Rastogi et al. 2001), cosmetics, and perfumes. In a 1996 study, a high proportion of perfumes and cosmetics labeled as containing natural ingredients in fact contained synthetic fragrances (Rastogi et al. 1996). Fragrances, particularly terpenes such as limonene, are associated with secondary chemical reactions in indoor air and can contribute to the production of formaldehyde, glycol ethers, ultrafine particles, and secondary organic aerosols (Nazaroff and Weschler 2004; Singer et al. 2006). Exposure to fragrances has been associated with a range of health effects, including allergic contact dermatitis, asthma and asthmatic exacerbations, headaches, and mucosal symptoms (Heydorn et al. 2003; Kumar et al. 1995; Steinemann 2009). Synthetic musks have been shown to have estrogenic effects (Bitsch et al. 2002; Schreurs et al. 2005; Seinen et al. 1999; van der Burg et al. 2008).

We frequently detected synthetic and natural fragrance chemicals in conventional samples. In our alternative products selected to allow only plant-based fragrance, synthetic fragrance chemicals were detected only in the facial cleanser, floor cleaner, and one sunscreen (< 100 µg/g). Bucinal, HHCB, and methyl ionone were the most frequently detected synthetic fragrance chemicals in conventional product samples. Concentrations of these and AHTN (acetyl hexamethyl tetralin), isobornyl acetate, and phenethyl alcohol included detects > 1,000 µg/g in fragrance/perfume, car and home air fresheners, and dryer sheets. Natural fragrance chemicals were common in conventional and alternative products. Most common natural fragrance chemicals included the terpenes limonene, hexyl cinnamal, and linalool. Concentrations of fragrance compounds were generally higher in conventional (21 instances > 1,000 µg/g) than in alternative products (2 instances > 1,000 µg/g), reflecting that product types used specifically to create scent (e.g., air freshener, perfume) were categorized as conventional and can be avoided altogether. We identified 26 alternative samples with no detectable fragrance chemicals.

Of the 34 conventional samples with detectable fragrance chemicals, 22 contained a product labeled with “fragrance” or other similar descriptors (e.g., “parfum”). Products that contained fragrance chemicals with no label indication were generally cleaners. Of the 17 alternative samples with detectable fragrance chemicals, 14 did not include “fragrance” or similar descriptors as ingredients. The other 3 were labeled “essential oil fragrance” or “plant based fragrance” and contained only natural fragrance compounds. Only 1 sample (sunscreen) of the 26 alternative samples with no detectable fragrance compounds had a reference to “fragrance” on the label, specifically listing “fragrance oil blend.”

*Glycol ethers.* Glycol ethers, a chemical class with > 80 compounds, are used in a broad array of cleaning applications because of their combined hydrophilic and lipophilic nature. They are often used in paints, varnishes, and cosmetics and have been detected in a variety of household products (Kwon et al. 2008; Plaisance et al. 2008). Biomonitoring methods are currently being developed, so large-scale studies are limited. In human studies, exposure to glycol ethers has been associated with low sperm mobility (Cherry et al. 2008), hematological effects (Starek et al. 2008), and asthma and allergies (Choi et al. 2010).

In the present study, we analyzed all samples for 2-butoxyethanol and 2,2-methoxyethoxyethanol, and in a later second sampling round, we analyzed 14 additional samples for six other glycol ethers. We detected glycol ethers in 3 conventional cleaners, face lotion, polish/wax, sunscreen, and in alternative shaving cream, pillow protector, and sunscreen samples. Of the 5 conventional samples with detectable 2-butoxyethanol, only the carpet cleaner was labeled as containing 2-butoxyethanol. When analyzed and detected, other glycol ethers were not listed on labels. Although we detected phenoxyethanol in conventional and alternative sunscreen samples, we did not detect this chemical in some conventional and alternative samples comprising products labeled as containing this compound; levels may have been < LOD.

*Cyclosiloxanes.* Cyclosiloxanes (cyclic volatile methylsiloxanes) are added to consumer products to enhance conditioning and spreading (Silicones Environmental, Health and Safety Council of North America 2011). Cyclosiloxanes are widely used, with the most common types being hexamethylcyclotrisiloxane, octamethylcyclotetrasiloxane (D_4_), D_5_, and dodecamethylcyclohexylsiloxane (D_6_). They have been found in cleaning products, personal care products, and baby products at concentrations as high as 1,010 µg/g (Horii and Kannan 2008; Wang et al. 2009). Cyclosiloxanes appear to be persistent and have relatively long half-lives in humans. D_4_ has weak estrogenic potential (Quinn et al. 2007) and D_5_ is potentially carcinogenic in rats (Wang et al. 2009).

Cyclosiloxanes were analyzed in 10 product types that were added during the second sampling round. All three cyclosiloxanes (D_4_, D_5_, and D_6_) were detected in the alternative composite sunscreen (D_5_ and D_6_ at > 4,000 µg/g) and in the conventional car interior cleaner (< 100 µg/g). One cyclosiloxane was detected in the conventional sunscreen (D_5_; 50 µg/g) and in the alternative shaving cream (D_6_; 10 µg/g). No product analyzed for cyclosiloxanes indicated “siloxane” on the label; however, two alternative sunscreens were labeled cyclomethicone, a generic name for polydimethylsiloxane, which includes D_4_, D_5_, and D_6_.

*UV filters.* Organic compounds that act as UV filters are added to many personal care products for skin protection and product stability. Three UV filters included in this study—benzophenone-3 (BP-3; oxybenzone), octyl dimethyl PABA (*p*-aminobenzoic acid), and octinoxate (octyl methoxycinnamate)—were detected in a previous study of 75 sunscreen products from European and U.S. manufacturers (Rastogi 2002). Biomonitoring data have indicated widespread exposure to some UV filters; BP-3 was detected in 96% of urine samples in NHANES 2003–2004 (Calafat et al. 2008a). Benzophenone-1, BP-3, and octinoxate are estrogenic *in vitro* and *in vivo* (Schlumpf et al. 2004) and act additively as mixtures (Kunz and Fent 2006).

We analyzed UV filters in sunscreens and eight other samples added during the second analytical round. We detected them at > 1% concentration in conventional and alternative sunscreen samples for which they were labeled as active ingredients. We detected lower concentrations of three UV filters in conventional sunscreen and shaving cream and in four of five alternative sunscreens, and none of these were labeled as containing these chemicals.

*Mixtures: chemicals that co-occur within and across products.* Our results show that one product can be a source of many chemicals of interest and that use of multiple products can result in exposure to an even larger number of chemicals.

We detected 0–22 analytes in a single product type (Figure 1). For composited samples, we do not know how many chemicals were in any one of the products; for alternative products, the number of detects ranged up to 11 analytes in shaving cream (Figure 2) and 17 in an individual sunscreen [see Supplemental Material, Figure S1 (http://dx.doi.org/10.1289/ehp.1104052)], illustrating the exposure to multiple compounds from a single product.

We identified chemicals that co-occur within a product type by estimating Kendall’s *tau* correlation coefficients [see Supplemental Material, Figure S2 (http://dx.doi.org/10.1289/​ehp.1104052)]. Many fragrance compounds were significantly correlated with each other and with DEP. For example, the natural fragrance limonene was correlated with natural fragrances linalool (τ_conventional_ = 0.43; τ_alternative_ = 0.59) and pinene (τ_conventional_ = 0.52; τ_alternative_ = 0.52) in both conventional and alternative samples. Limonene and linalool also were positively correlated with DEP in both conventional and alternative samples (τ = 0.31–0.52). In the conventional samples, DEP was positively correlated with several fragrance compounds (AHTN, benzyl acetate, bucinal, hexyl cinnemal, HHCB, linalool, limonene, and methyl ionone; τ = 0.34–0.56), which supports the idea that DEP is a common carrier for fragrances. The finding of positive correlations among the fragrance compounds may be influenced by compositing. For example, if each individual product within a product type uses a different fragrance, these compounds will be correlated in the composites, even though an individual product may contain only one of the compounds. We also found that 4-*t*-nonylphenol and DEHP were correlated in conventional samples (τ = 0.4), consistent with use of both compounds as plastics additives. Nonylphenol monoethoxylate and nonylphenol diethoxylate were positively correlated (τ_conventional_ = 0.35; *p* = 0.1), consistent with their presence in commercial APEO mixtures. In alternative samples, methyl paraben was positively correlated with all three cyclosiloxanes (τ = 0.69–0.87), and the cyclosiloxanes were positively correlated with each other (τ = 0.62–0.73).

Our results also indicate that use of multiple products can lead to exposure to an even larger mixture of compounds, even if a consumer selected products considered alternative according to our criteria. For example, a consumer who used the alternative surface cleaner, tub and tile cleaner, laundry detergent, bar soap, shampoo and conditioner, facial cleanser and lotion, and toothpaste (a plausible array of product types for an individual) would potentially be exposed to at least 19 compounds: two parabens, three phthalates, MEA, DEA, five alkylphenols, and seven fragrances.

The impact of exposures via one product or multiple products is of concern because of the potential combined effects of EDCs or compounds associated with asthma. Our analysis demonstrates that chemical combinations are common in consumer products, and results highlight combinations for toxicity testing, risk assessment, and epidemiological study. Toxicity testing can identify common modes of action for co-occurring chemicals, and risk assessment can then investigate cumulative exposures to multiple chemicals. Considering effects of co-occurring compounds in risk assessment would advance the recommendations of the National Research Council (2008). Similarly, in epidemiological studies, co-occurring exposures need to be understood together, because they may have additive or interacting effects or result in confounding. As an example of possible confounding, several studies have shown an association of endocrine-related health effects with DEP, which does not show activity in animal studies (Duty et al. 2003; Hauser et al. 2007; López-Carrillo et al. 2010; Swan et al. 2005); instead, DEP could be a marker for a large number of synthetic and natural fragrances, which do have activity. This suggests an important area for future research is to characterize the endocrine activity of fragrances and to measure these compounds in epidemiological studies. Epidemiological studies should include collaborations with toxicologists to help design and interpret findings.

*Variability within product types.* Although our study was not designed to focus on variability in the composition of different individual products within a type, we examined this question for sunscreens. Our study provides some information about how exposure may differ depending on brand selection and allows us to investigate the effect of compositing, which is a limitation.

Using sunscreens as an example, we observed substantial variability in composition of products within this product type [see Supplemental Material, Figure S1 (http://dx.doi.org/10.1289/ehp.1104052)]. Among the 5 alternative sunscreens, we detected 4–17 compounds per sample, with a total of 24 chemicals detected in the sunscreens. The product with the highest number of detects was marketed for children and favorably rated by a popular environmental health site. The variable composition of individual products within a class is important to consider in exposure modeling and in epidemiological studies that rely on self-reported product use as a proxy for exposure.

*Limitations.* To our knowledge, this study is one of the first to look for a large and varied suite of compounds in a broad range of product types; however, the product types and chemicals we included are still a small fraction of those in use, so this report is not comprehensive. In addition, the alternative and conventional products in this study may not be representative. In particular, alternative products, selected according to criteria in Table 1, were mostly purchased at one store with its own criteria, and we do not know how these criteria influenced our product selection. All products were purchased in 2007 and 2008; because formulations may have been changed, products purchased today could be different.

We chose to composite conventional products to increase representativeness and limit analytical costs; however, this strategy limits interpretation in several ways. First, compositing does not allow observation of extreme high and low concentrations because it is meant to optimize the estimate of the average concentration. Second, compositing may increase or decrease the number of compounds detected. Twelve chemical concentrations in individual sunscreen samples were diluted to lower concentration categories in the calculated composite, including to values < LOD [see Supplemental Material, Figure S1 (http://dx.doi.org/10.1289/ehp.1104052)]. Conversely, the number of detects could increase if manufacturers use different chemicals to achieve a particular function in the product (e.g., scent), thereby increasing the number of different chemicals in a composite. To evaluate the effects of compositing on number of detects, we calculated composites from varying numbers of individual sunscreens. The number of detected chemicals in possible sunscreen composites ranged from 5 to 21 [see Supplemental Material, Figure S3 (http://dx.doi.org/10.1289/ehp.1104052)] and was positively correlated with the number of products in the composite (see Supplemental Material, Figure S4). These results indicate the varying chemical formulations within a product type. Finally, because we composited conventional and not alternative products and the composites comprise varying numbers of products per sample, direct comparisons between conventional and alternative products and some comparisons between conventional product types could be misleading.

## Conclusions

We tested an exceptionally wide range of products, including 50 types of personal care and cleaning products as well as selected household goods, for 66 compounds identified as EDCs or asthma related. We detected 55 compounds, suggesting a wide range of exposures from common products. Results suggest that vinyl products are an important source of DEHP in homes. In other products, the highest concentrations and numbers of detects were in fragranced products (e.g., perfume, air fresheners, and dryer sheets) and sunscreen. To our knowledge, this is the first test of sunscreens for a range of EDCs. In addition to the labeled ingredients, sunscreens contained up to seven target chemicals that were not included on the product label. The highest number of detects in sunscreen was in a product favorably rated by a popular environmental health website and marketed for babies, children, and sensitive adults; this illustrates the limitations of rating products based on ingredients disclosed on product labels. In addition to a broad assessment of chemicals in widely used personal care and cleaning products, one of our goals was to identify a strategy for reducing exposure by removing or substituting products. Our shopping criteria did identify a set of alternative products containing no BPA or antimicrobials and limited synthetic fragrance. We detected DCP, DINP, and DPP only in alternative products, suggesting the possibility that manufacturers have substituted these antiandrogenic phthalates for the better known and also antiandrogenic phthalates DEHP, DBP, and BBP, which are common in conventional products. Our observations of multiple chemicals of concern in composites of high-market-share products coupled with consumers’ use of multiple product types (e.g., laundry detergent plus dish soap plus shampoo) highlight the importance of considering the cumulative toxicological effects of combined exposures. Our correlation analysis identifies mixtures for evaluation and also raises caution that associations in epidemiological studies may be due to unmeasured chemicals that co-occur with the study target. Disclosure of product ingredients would enable researchers to identify exposures for study and risk evaluation and allow consumers to make decisions consistent with their personal values.

## Supplemental Material

(848 KB) PDF

## References

[r1] Adolfsson-Erici M, Pettersson M, Parkkonen J, Sturve J. (2002). Triclosan, a commonly used bactericide found in human milk and in the aquatic environment in Sweden.. Chemosphere.

[r2] Association of Occupational and Environmental Clinics (2010). Description of the AOEC Exposure Code System.. http://www.aoecdata.org/.

[r3] Bickers DR, Calow P, Greim HA, Hanifin JM, Rogers AE, Saurat JH (2003). The safety assessment of fragrance materials.. Regul Toxicol Pharmacol.

[r4] Bitsch N, Dudas C, Körner W, Failing K, Biselli S, Rimkus G (2002). Estrogenic activity of musk fragrances detected by the E-Screen assay using human MCF-7 cells.. Arch Environ Contam Toxicol.

[r5] Boberg J, Christiansen S, Axelstad M, Kledal TS, Vinggaard AM, Dalgaard M (2011). Reproductive and behavioral effects of diisononyl phthalate (DINP) in perinatally exposed rats.. Reprod Toxicol.

[r6] Bonefeld-Jørgensen EC, Long M, Hofmeister MV, Vinggaard AM (2007). Endocrine-disrupting potential of bisphenol A, bisphenol A dimethacrylate, 4-*n*-nonylphenol, and 4-*n*-octylphenol *in vitro*: new data and a brief review.. Environ Health Perspect.

[r7] Bornehag CG, Nanberg E (2010). Phthalate exposure and asthma in children.. Int J Androl.

[r8] Bornehag CG, Sundell J, Weschler CJ, Sigsgaard T, Lundgren B, Hasselgren M (2004). The association between asthma and allergic symptoms in children and phthalates in house dust: a nested case–control study.. Environ Health Perspect.

[r9] Bridges B. (2002). Fragrance: emerging health and environmental concerns.. Flavour Fragr J.

[r10] Brody JG, Morello-Frosch R, Zota A, Brown P, Perez C, Rudel RA (2009). Linking exposure assessment science with policy objectives for environmental justice and breast cancer advocacy: the northern California household exposure study.. Am J Public Health.

[r11] Calafat AM, Wong LY, Ye X, Reidy JA, Needham LL (2008a). Concentrations of the sunscreen agent benzophenone-3 in residents of the United States: National Health and Nutrition Examination Survey 2003–2004.. Environ Health Perspect.

[r12] Calafat AM, Ye X, Wong LY, Reidy JA, Needham LL (2008b). Exposure of the U.S. population to bisphenol A and 4-*tertiary*-octylphenol: 2003–2004.. Environ Health Perspect.

[r13] Calafat AM, Ye X, Wong LY, Reidy JA, Needham LL (2008c). Urinary concentrations of triclosan in the U.S. population: 2003–2004.. Environ Health Perspect.

[r14] CDC (Centers for Disease Control and Prevention) (2009). Fourth National Report on Human Exposure to Environmental Chemicals.. http://www.cdc.gov/ExposureReport/pdf/FourthReport.pdf.

[r15] Chen J, Ahn KC, Gee NA, Ahmed MI, Duleba AJ, Zhao L (2008). Triclocarban enhances testosterone action: a new type of endocrine disruptor?. Endocrinology.

[r16] Cherry N, Moore H, McNamee R, Pacey A, Burgess G, Clyma JA (2008). Occupation and male infertility: glycol ethers and other exposures.. Occup Environ Med.

[r17] Choi H Schmidbauer N Sundell J Hasselgren M Spengler JD Bornehag CG 2010 Common household chemicals and the allergy risks in pre-school age children. PLoS ONE 5 10 e13423; doi:10.1371/journal.pone.0013423 [Online 18 October 2010] 20976153 PMC2956675

[r18] Colborn T, vom Saal FS, Soto A (1993). Developmental effects of endocrine-disrupting chemicals in wildlife and humans.. Environ Health Perspect.

[r19] Dodson RE, Levy JI, Spengler JD, Shine JP, Bennett DH (2008). Influence of basements, garages, and common hallways on indoor residential volatile organic compound concentrations.. Atmos Environ.

[r20] Douwes J, Pearce N. (2002). Asthma and the westernization ‘package.’. Int J Epidemiol.

[r21] Dunagan SC, Dodson RE, Rudel RA, Brody JG (2011). Toxics use reduction in the home: lessons learned from household exposure studies.. J Clean Prod.

[r22] Duty SM, Singh NP, Silva MJ, Barr DB, Brock JW, Ryan L (2003). The relationship between environmental exposures to phthalates and DNA damage in human sperm using the neutral comet assay.. Environ Health Perspect.

[r23] Engel SM, Miodovnik A, Canfield RL, Zhu C, Silva MJ, Calafat AM (2010). Prenatal phthalate exposure is associated with childhood behavior and executive functioning.. Environ Health Perspect.

[r24] Environmental Working Group (2011). EWG’s Skin Deep Cosmetics Database.. http://www.ewg.org/skindeep/.

[r25] European Commission (2002). European Union Risk Assessment Report: 4-Nonylphenol (Branched) and Nonylphenol. EUR 20387 EN. Luxembourg:Joint Research Centre Institute for Health and Consumer Protection, European Chemicals Bureau.. http://www.bfr.bund.de/cm/343/4_nonylphenol_und_nonylphenol.pdf.

[r26] European Commission (2011). CosIng.. http://ec.europa.eu/consumers/cosmetics/cosing.

[r27] Fair Packaging and Labeling Act (1967). Public Law 89-755.

[r28] FDA (Food and Drug Administration) (2009). Cosmetic Labeling Manual.. http://www.fda.gov/Cosmetics/CosmeticLabelingLabelClaims/CosmeticLabelingManual/default.htm.

[r29] Federal Food Drug, Act Cosmetic (1938). Public Law.

[r30] FIFRA (Federal Insecticide, Fungicide, and Rodenticide Act) (1972).

[r31] Food and Agriculture Organization of the United Nations/World Health Organization) (2010). Joint FAO/WHO Expert Meeting to Review Toxicological and Health Aspects of Bisphenol A: Summary Report including Report of Stakeholder Meeting on Bisphenol A.. http://www.who.int/foodsafety/chem/chemicals/BPA_Summary2010.pdf.

[r32] GoodGuide (2012). GoodGuide Homepage.. http://www.goodguide.com.

[r33] Hannas BR, Furr J, Lambright CS, Wilson VS, Foster PM, Gray LE (2011). Dipentyl phthalate dosing during sexual differentiation disrupts fetal testis function and postnatal development of the male Sprague-Dawley rat with greater relative potency than other phthalates.. Toxicol Sci.

[r34] Hauser R, Calafat AM (2005). Phthalates and human health.. Occup Environ Med.

[r35] Hauser R, Meeker JD, Duty S, Silva MJ, Calafat AM (2006). Altered semen quality in relation to urinary concentrations of phthalate monoester and oxidative metabolites.. Epidemiology.

[r36] Hauser R, Meeker JD, Singh NP, Silva MJ, Ryan L, Duty S (2007). DNA damage in human sperm is related to urinary levels of phthalate monoester and oxidative metabolites.. Hum Reprod.

[r37] Heindel JJ, Gulati DK, Mounce RC, Russell SR, Lamb JC (1989). Reproductive toxicity of three phthalic acid esters in a continuous breeding protocol.. Fundam Appl Toxicol.

[r38] Henley DV, Lipson N, Korach KS, Bloch CA (2007). Prepubertal gynecomastia linked to lavender and tea tree oils.. N Engl J Med.

[r39] Heudorf U, Mersch-Sundermann V, Angerer E. (2007). Phthalates: toxicology and exposure.. Int J Hyg Environ Health.

[r40] Heydorn S, Johansen JD, Andersen KE, Bruze M, Svedman C, White IR (2003). Fragrance allergy in patients with hand eczema—a clinical study.. Contact Dermatitis.

[r41] Horii Y, Kannan K. (2008). Survey of organosilicone compounds, including cyclic and linear siloxanes, in personal-care and household products.. Arch Environ Contam Toxicol.

[r42] Howdeshell KL, Wilson VS, Furr J, Lambright CR, Rider CV, Blystone CR (2008). A mixture of five phthalate esters inhibits fetal testicular testosterone production in the Sprague-Dawley rat in a cumulative, dose-additive manner.. Toxicol Sci.

[r43] Hubinger JC, Havery DC (2006). Analysis of consumer cosmetic products for phthalate esters.. J Cosmet Sci.

[r44] IARC (International Agency for Research on Cancer) (1999). Some Chemicals that Cause Tumours of the Kidney or Urinary Bladder in Rodents and Some Other Substances. IARC Monogr Eval Carcinog Risk Hum 73:1–641.. http://monographs.iarc.fr/ENG/Monographs/vol73/mono73.pdf.

[r45] IARC (International Agency for Research on Cancer) (2000). Some Industrial Chemicals. IARC Monogr Eval Carcinog Risk Hum 77:1–529.. http://monographs.iarc.fr/ENG/Monographs/vol77/mono77.pdf.

[r46] International Fragrance Association (2010). Ingredients.. http://www.ifraorg.org/en-us/Ingredients_2.

[r47] Jie X, Yang W, Jie Y, Hashim JH, Liu XY, Fan QY (2010). Toxic effect of gestational exposure to nonylphenol on F1 male rats.. Birth Defects Res B Dev Reprod Toxicol.

[r48] Kamijo Y, Hayashi I, Ide A, Yoshimura K, Soma K, Majima M. (2009). Effects of inhaled monoethanolamine on bronchoconstriction.. J Appl Toxicol.

[r49] Kang KS, Che JH, Ryu DY, Kim TW, Li GX, Lee YS (2002). Decreased sperm number and motile activity on the F1 offspring maternally exposed to butyl *p*-hydroxybenzoic acid (butyl paraben).. J Vet Med Sci.

[r50] Kimber I, Dearmna RJ (2010). An assessment of the ability of phthalates to influence immune and allergic responses.. Toxicology.

[r51] Koniecki D, Wang R, Moody RP, Zhu J (2011). Phthalates in cosmetic and personal care products: concentrations and possible dermal exposure.. Environ Res.

[r52] Kumar P, Caradonna-Graham VM, Gupta S, Cai X, Rao PN, Thompson J (1995). Inhalation challenge effects of perfume scent strips in patients with asthma.. Ann Allergy Asthma Immunol.

[r53] Kunz P, Fent K. (2006). Estrogenic activity of UV filter mixtures.. Toxicol Appl Pharmacol.

[r54] Kwon KD, Jo WK, Lim HJ, Jeong WS (2008). Volatile pollutants emitted from selected liquid household products.. Environ Sci Pollut Res Int.

[r55] López-Carrillo L, Hernández-Ramírez RU, Calafat AM, Torres-Sánchez L, Galván-Portillo M, Needham LL (2010). Exposure to phthalates and breast cancer risk in northern Mexico.. Environ Health Perspect.

[r56] Lorber M. (2008). Exposure of Americans to polybrominated diphenyl ethers.. J Expo Sci Environ Epidemiol.

[r57] Lu CS, Toepel K, Irish R, Fenske RA, Barr DB, Bravo R (2006). Organic diets significantly lower children’s dietary exposure to organophosphorus pesticides.. Environ Health Perspect.

[r58] Mäkelä R, Kauppi P, Suuronen K, Tuppurainen M, Hannu T. (2011). Occupational asthma in professional cleaning work: a clinical study.. Occup Med (Lond).

[r59] Meeker JD, Calafat AM, Hauser R (2009a). Urinary metabolites of di(2-ethylhexyl) phthalate are associated with decreased steroid hormone levels in adult men.. J Androl.

[r60] Meeker JD, Sathyanarayana S, Swan SH (2009b). Phthalates and other additives in plastics: human exposure and associated health outcomes.. Philos Trans R Soc Lond B Biol Sci.

[r61] Mendiola J Meeker JD Jorgensen N Andersson AM Liu F Calafat AM 2011 Urinary concentrations of di(2-ethylhexyl) phthalate metabolites and serum reproductive hormones: Pooled analysis of fertile and infertile men. J Androl; doi:10.2164/jandrol.111.013557 [19 May 2011] PMC3433231 21597090

[r62] National Library of Medicine (2010a). TOXNET.. http://toxnet.nlm.nih.gov.

[r63] National Library of Medicine (2010b). Household Products Database.. http://householdproducts.nlm.nih.gov.

[r64] Nazaroff WW, Weschler CJ (2004). Cleaning products and air fresheners: exposure to primary and secondary air pollutants.. Atmos Environ.

[r65] Newton E, Rudel R. (2007). Estimating correlation with multiply censored data arising from the adjustment of singly censored data.. Environ Sci Technol.

[r66] National Research Council (2008). Phthalates and Cumulative Risk Assessment: The Task Ahead.

[r67] Paul KB, Hedge JM, DeVito MJ, Crofton KM (2010). Short-term exposure to triclosan decreases thyroxine *in vivo* via upregulation of hepatic catabolism in young Long-Evans rats.. Toxicol Sci.

[r68] Perencevich EN, Wong MT, Harris AD (2001). National and regional assessment of the antibacterial soap market: a step toward determining the impact of prevalent antibacterial soaps.. Am J Infect Control.

[r69] Piipari R, Tuppurainen M, Tuomi T, Mäntylä L, Henriks-Eckerman ML, Keskinen H (1998). Diethanolamine-induced occupational asthma, a case report.. Clin Exp Allergy.

[r70] Plaisance H, Desmettres P, Leonardis T, Pennequin-Cardinal A, Locoge N, Galloo JC (2008). Passive sampling of glycol ethers and their acetates in indoor air.. J Environ Monit.

[r71] Quinn AL, Regan JM, Tobin JM, Marinik BJ, McMahon JM, McNett DA (2007). *In vitro* and *in vivo* evaluation of the estrogenic, androgenic, and progestagenic potential of two cyclic siloxanes.. Toxicol Sci.

[r72] Rakkestad KE, Dye CJ, Yttri KE, Holme JA, Hongslo JK, Schwarze PE (2007). Phthalate levels in Norwegian indoor air related to particle size fraction.. J Environ Monit.

[r73] Rastogi SC (2002). UV filters in sunscreen products—a survey.. Contact Dermatitis.

[r74] Rastogi SC, Heydorn S, Johansen JD, Basketter DA (2001). Fragrance chemicals in domestic and occupational products.. Contact Dermatitis.

[r75] Rastogi SC, Johansen JD, Menné T (1996). Natural ingredients based cosmetics. Content of selected fragrance sensitizers.. Contact Dermatitis.

[r76] Rastogi SC, Schouten A, de Kruijf N, Weijland JW (1995). Contents of methyl-, ethyl-, propyl-, butyl- and benzylparaben in cosmetic products.. Contact Dermatitis.

[r77] Ravid U, Elkabetz M, Zamir C, Cohen K, Larkov O, Aly R. (2010). Authenticity assessment of natural fruit flavour compounds in foods and beverages by auto-HS–SPME stereoselective GC–MS.. Flavour Fragr J.

[r78] Reiner JL, Kannan K (2006). A survey of polycyclic musks in selected household commodities from the United States.. Chemosphere.

[r79] Rodríguez PE, Sanchez MS (2010). Maternal exposure to triclosan impairs thyroid homeostasis and female pubertal development in Wistar rat offspring.. J Toxicol Environ Health A.

[r80] Rotroff DM, Wetmore BA, Dix DJ, Ferguson SS, Clewell HJ, Houck KA (2010). Incorporating human dosimetry and exposure into high-throughput *in vitro* toxicity screening.. Toxicol Sci.

[r81] Routledge EJ, Parker J, Odum J, Ashby J, Sumpter JP (1998). Some alkyl hydroxy benzoate preservatives (parabens) are estrogenic.. Toxicol Appl Pharmacol.

[r82] Rudel RA, Camann DE, Spengler JD, Korn LR, Brody JG (2003). Phthalates, alkylphenols, pesticides, polybrominated diphenyl ethers, and other endocrine-disrupting compounds in indoor air and dust.. Environ Sci Technol.

[r83] Rudel RA, Dodson RE, Perovich LJ, Morello-Frosch R, Camann DE, Zuniga MM (2010). Semivolatile endocrine-disrupting compounds in paired indoor and outdoor air in two northern California communities.. Environ Sci Technol.

[r84] Rudel RA, Gray JM, Engel CL, Rawsthorne TW, Dodson RE, Ackerman JM (2011). Food packaging and bisphenol A and bis(2-ethyhexyl) phthalate exposure: findings from a dietary intervention.. Environ Health Perspect.

[r85] Rudel RA, Perovich LJ (2009). Endocrine disrupting chemicals in indoor and outdoor air.. Atmos Environ.

[r86] Rudel RA Seryak LM Brody JG 2008 PCB-containing wood floor finish is a likely source of elevated PCBs in residents’ blood, household air and dust: a case study of exposure. Environ Health 7 2; doi:10.1186/1476-069X-7-2 [Online 17 January 2008] 18201376 PMC2267460

[r87] Saillenfait AM, Gallissot F, Sabate JP (2009). Differential developmental toxicities of di-*n*-hexyl phthalate and dicyclohexyl phthalate administered orally to rats.. J Appl Toxicol.

[r88] Savonius B, Keskinen H, Tuppurainen M, Kanerva L. (1994). Occupational asthma caused by ethanolamines.. Allergy.

[r89] Schlumpf M, Schmid P, Durrer S, Conscience M, Maerkel K, Henseler M (2004). Endocrine activity and developmental toxicity of cosmetic UV filters—an update.. Toxicology.

[r90] Schreurs RH, Sonneveld E, Jansen JH, Seinen W, van der Burg B (2005). Interaction of polycyclic musks and UV filters with the estrogen receptor (ER), androgen receptor (AR), and progesterone receptor (PR) in reporter gene bioassays.. Toxicol Sci.

[r91] Seinen W, Lemmen JG, Pieters RHH, Verbruggen EMJ, van der Burg B (1999). AHTN and HHCB show weak estrogic —but no uterotrophic activity.. Toxicol Lett.

[r92] Shen H-Y, Jiang H-L, Mao H-L, Pan G, Zhou L, Cao Y-F (2007). Simultaneous determination of seven phthalates and four parabens in cosmetic products using HPLC-DAD and GC-MS methods.. J Sep Sci.

[r93] Silent Spring Institute (2012). Table: Consumer Products Tested for Endocrine Disruptors and Asthma-Associated Chemicals.. http://silentspring.org/table-consumer-products-tested-endocrine-disruptors-and-asthma-associated-chemicals.

[r94] Silicones Environmental, Health and Safety Council of North America (2011). Science, Health and Safety: Decamethylcyclopentasiloxane (D_5_).. http://www.sehsc.com/d5.asp.

[r95] Singer BC, Coleman BK, Destaillats H, Hodgson AT, Lunden MM, Weschler CJ (2006). Indoor secondary pollutants from cleaning product and air freshener use in the presence of ozone.. Atmos Environ.

[r96] Smith SW (2009). Chiral toxicology: it’s the same thing...only different.. Toxicol Sci.

[r97] Soni MG, Burdock GA, Taylor SL, Greenberg NA (2001). Safety assessment of propyl paraben: a review of the published literature.. Food Chem Toxicol.

[r98] Starek A, Szymczak W, Zapor L. (2008). Hematological effects of four ethylene glycol monoalkyl ethers in short-term repeated exposure in rats.. Arch Toxicol.

[r99] Steinemann AC (2009). Fragranced consumer products and undisclosed ingredients.. Environ Impact Assess Rev.

[r100] Steinemann AC, MacGregor IC, Gordon SM, Gallagher LG, Davis AL, Ribeiro DS (2011). Fragranced consumer products: chemicals emitted, ingredients unlisted.. Environ Impact Assess Rev.

[r101] Stoker TE, Gibson EK, Zorrilla LM (2010). Triclosan exposure modulates estrogen-dependent responses in the female Wistar rat.. Toxicol Sci.

[r102] Swan SH (2008). Environmental phthalate exposure in relation to reproductive outcomes and other health endpoints in humans.. Environ Res.

[r103] Swan SH, Main KM, Liu F, Stewart SL, Kruse RL, Calafat AM (2005). Decrease in anogenital distance among male infants with prenatal phthalate exposure.. Environ Health Perspect.

[r104] van der Burg B, Schreurs R, van der Linden S, Seinen W, Brouwer A, Sonneveld E. (2008). Endocrine effects of polycyclic musks: do we smell a rat?. Int J Androl.

[r105] Wang R, Moody RP, Koniecki D, Zhu J (2009). Low molecular weight cyclic volatile methylsiloxanes in cosmetic products sold in Canda: implications for dermal exposure.. Environ Int.

[r106] Weschler CJ (2009). Changes in indoor pollutants since the 1950s.. Atmos Environ.

[r107] Ye X, Bishop AM, Reidy JA, Needham LL, Calafat AM (2006). Parabens as urinary biomarkers of exposure in humans.. Environ Health Perspect.

[r108] Zhang XL, Yao Y, Zeng XY, Qian GR, Guo YW, Wu MH (2008). Synthetic musks in the aquatic environment and personal care products in Shanghai, China.. Chemosphere.

[r109] Zota AR, Rudel RA, Morello-Frosch RA, Brody JG (2008). Elevated house dust and serum concentrations of PBDEs in California: unintended consequences of furniture flammability standards?. Environ Sci Technol.

