# Supplementary material for: Endocrine Disruptors and Asthma-Associated Chemicals in Consumer Products
Source: Environ Health Perspect. 2012 Mar 8;120(7):935–43. doi: 10.1289/ehp.1104052 (PMC3404651; doi:10.1289/ehp.1104052)
Supplement: (848 KB) PDF [file ehp.1104052.s001.pdf]

SUPPLEMENTAL MATERIAL
FOR

Endocrine Disruptors and Asthma-Associated Chemicals in Consumer Products

Robin E. Dodson,[1]* Marcia Nishioka,[2] Laurel J. Standley,[1,3] Laura J. Perovich,[1] Julia G. Brody,[1] and Ruthann A. Rudel[1]

[1] Silent Spring Institute, Newton, MA 02458
[2] Battelle Memorial Institute, Columbus, OH 43201
[3] Clear Current, LLC, Belmont, CA 94002

CONTENTS:

S-2 Table S1. Chemical class summaries, including uses, potential health effects, and compounds analyzed

S-5 Figure S1. Concentrations of target compounds in sunscreen samples

S-6 Figure S2. Correlation matrix for conventional and alternative products

S-7 Figure S3. Effects of compositing products on detection patterns: An example using 5 alternative sunscreens.

S-8 Figure S4. Number of products per composite versus number of detected chemicals for conventional samples.

S-9 Analytical Quality Assurance/Quality Control (QA/QC) Methods and Results

S-11 Table S2. Summary statistics for solvent method blanks (μg) in each analytical round.

S-13 Table S3. Summary statistics for precision, calculated as relative percent difference (%)

S-15 Table S4. Summary statistics for recoveries (%) from spiked products

S-17 Figure S5. Surrogate recovery distributions.

S-18 References cited in Supplemental Material

Table S1. Chemical class summaries, including uses, potential health effects, and compounds analyzed.

Chemical Class	Use(s) in Products[a]	Potential Health Concerns[b]	Chemicals
parabens	preservative; anti-microbial agent	endocrine disruption (Kang et al. 2002)	methyl paraben<br>ethyl paraben<br>butyl paraben
phthalates	plastic additives; solvents in cosmetics and perfumes; inert ingredient in pesticides	endocrine disruption (Hannas et al. 2011; Hauser et al. 2006; Heindel et al. 1989; Howdeshell et al. 2008; Meeker et al. 2009; Mendiola et al. 2011; Swan et al. 2005).<br>asthma associated (Bornehag et al. 2004; Bornehag and Nanberg 2010)	bis(2-ethylhexyl) adipate<br>bis(2-ethyhexyl) phthalate<br>benzylbutyl phthalate<br>*di-amyl phthalate*<br>di-cyclohexyl phthalate<br>di-isobutyl phthalate<br>di-isononyl phthalate<br>di-n-butylphthalate<br>di-n-hexyl phthalate<br>di-n-octyl phthalate<br>di-n-propyl phthalate<br>diethyl phthalate
bisphenol A	production of polycarbonate plastic and epoxy resins	endocrine disruption (FAO/WHO 2010; NTP-CERHR 2008)	bisphenol A
antimicrobials	anti-microbial agent	endocrine disruption (Chen et al. 2008; Orton et al. 2011; Stoker et al. 2010)	*1,4-dichlorobenzene*<br>*o-phenylphenol*<br>triclosan<br>triclocarban
ethanolamines	solvent in cleaners; emulsifier in creams and lotions	asthma associated (Kamijo et al. 2009; Makela et al. 2011; Piipari et al. 1998; Savonius et al. 1994)	monoethanolamine<br>diethanolamine
alkylphenols	surfactant; disinfectant; inert ingredient in pesticides	endocrine disruption (Jie et al. 2010)	4-t-octylphenol<br>octylphenol monoethoxylate<br>octylphenol diethoxylate<br>4-t-nonylphenol<br>nonylphenol monoethoxylate<br>nonylphenol diethoxylate

Chemical Class	Use(s) in Products[a]	Potential Health Concerns[b]	Chemicals
fragrances	scent; masking agent	endocrine disruption (Bitsch et al. 2002; Schreurs et al. 2005; Seinen et al. 1999; van der Burg et al. 2008)<br>asthma associated (Kumar et al. 1995)	natural[c]<br>benzyl acetate<br>eugenol<br>hexyl cinnemal<br>limonene<br>linalool<br>methyl eugenol<br>methyl salicylate<br>pinene<br>terpineol<br>synthetic<br>AHTN<br>bucinal<br>diphenyl ether<br>DPMI<br>HHCB<br>isobornyl acetate<br>methyl ionone<br>musk ketone<br>musk xylene<br>phenethyl alcohol
glycol ethers	solvent	asthma associated (Choi et al. 2010)	*2-isopropoxyethanol (R2)*<br>*2-propoxyethanol (R2)*<br>2-butoxylethanol<br>2-phenoxylethanol (R2)<br>*2-benzyloxyethanol (R2)*<br>2,2-methoxyethoxyethanol<br>*2,2-ethyoxyethoxyethanol (R2)*<br>2,2-butoxyethoxyethanol (R2)
perfluorinated	stain resistance	endocrine disruption (White et al. 2011)	*8:2 FTOH*
cyclosiloxanes	enhance conditioning and spreading	endocrine disruption (Quinn et al. 2007)<br>carcinogenicitiy (Wang et al. 2009)	octamethylcyclotetrasiloxane (D4) (R2)<br>decamethylcyclopentasiloxane (D5) (R2)<br>dodecamethylcyclohexylsiloxane (D6) (R2)

Chemical Class	Use(s) in Products[a]	Potential Health Concerns[b]	Chemicals
UV filters	skin protection; product stability and durability	endocrine disruption (Schlumpf et al. 2004)	*3,4-methylbenzylidene camphor (R2)*<br>benzophenone (R2)<br>benzophenone-1 (R2)<br>*benzophenone-2 (R2)*<br>benzophenone-3 (R2)<br>oxtinoxate (R2)<br>*octadimethyl PABA (R2)*

[a] General use categories obtained from the NLM Hazardous Substance Data Bank and/or scientific literature
[b] Health effects have not necessarily been reported for all chemicals within the chemical class. Among the EDCs in this study, phthalates are the only chemical group for which there is supporting evidence of health effects from human studies. All asthma-associations are derived from human studies.

[c] Natural fragrances are readily available from plant materials, but can also be synthesized. Stereoisomer composition will differ for chemically synthesized materials. Our analysis did not determine whether these were synthesized or derived from plant materials.

R2 indicates chemicals added during the second round of sampling
Italicized chemicals were not detected in any product

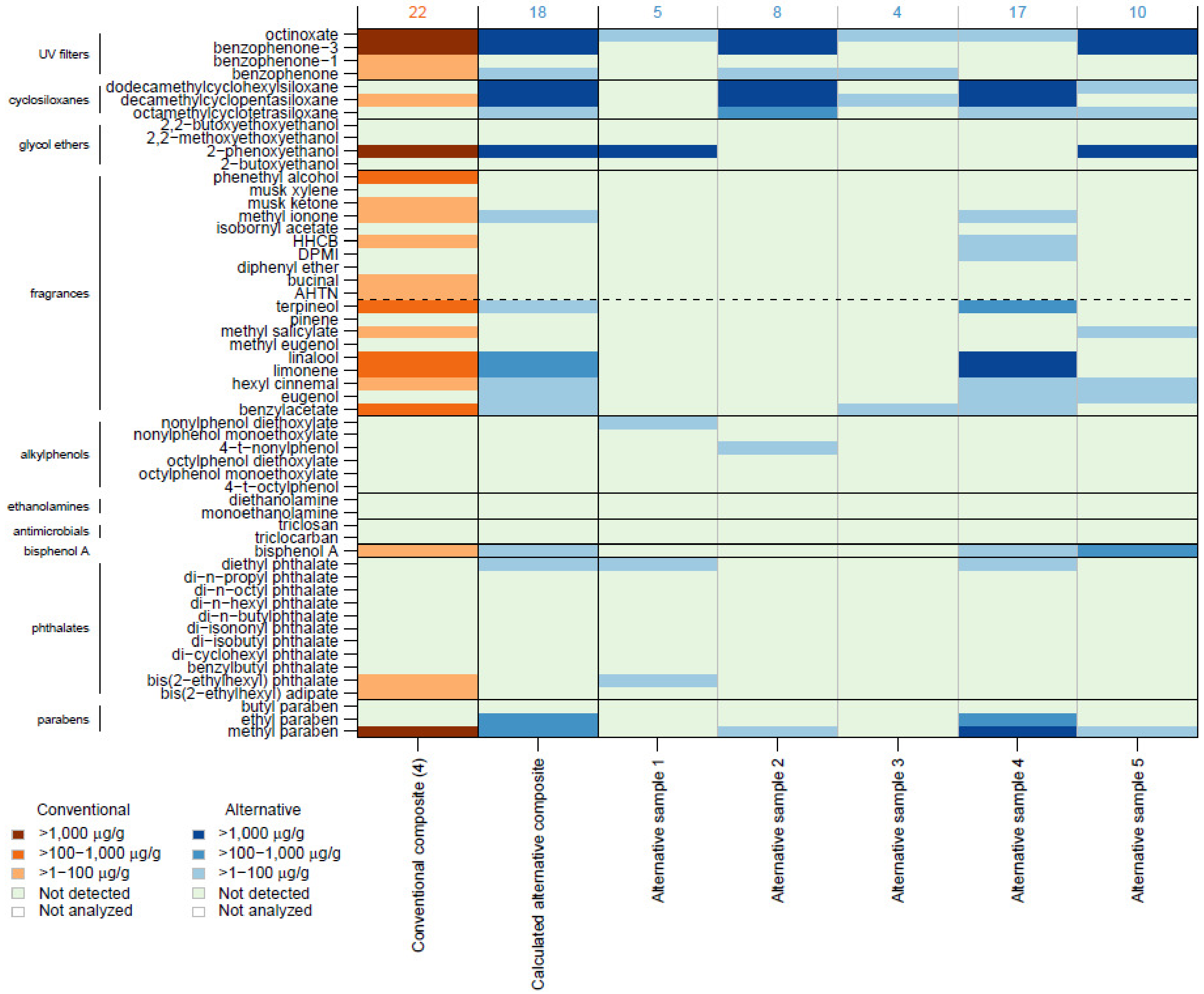

Figure S1. Concentrations of target compounds in sunscreen samples. Compounds are grouped by chemical class, with natural and synthetic fragrances distinguished by a dashed horizontal line. Horizontal (x-axis) shows the conventional sunscreen sample, which was a composite of 4 sunscreens; the calculated composite of 5 alternative sunscreens that were analyzed individually; and then results for each of the 5 alternative sunscreens. Numbers in the top margin count the number of chemicals detected in each sample; numbers in the right margin count number of products containing each compound.

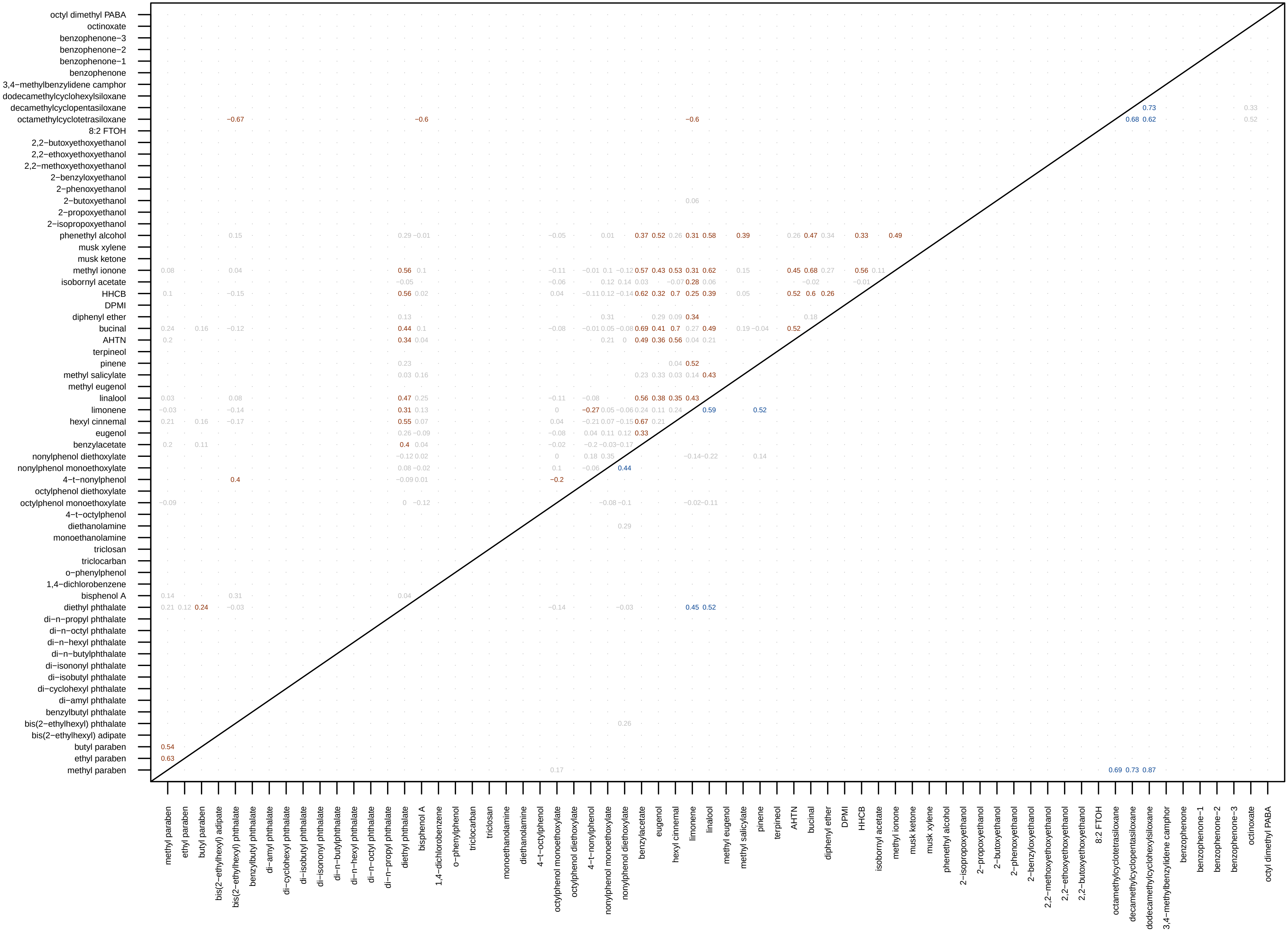

Figure S2. Kendall's tau correlation estimates for concentrations in conventional (upper left; red) and alternative (lower right; blue) products. Significant correlation estimates (p<0.05) in color. '.' indicates insufficient number of detected pairs for correlation analysis.Compounds are sorted by chemical class and match other figures and tables. See the Mixtures section in the manuscript for a discussion of the results.

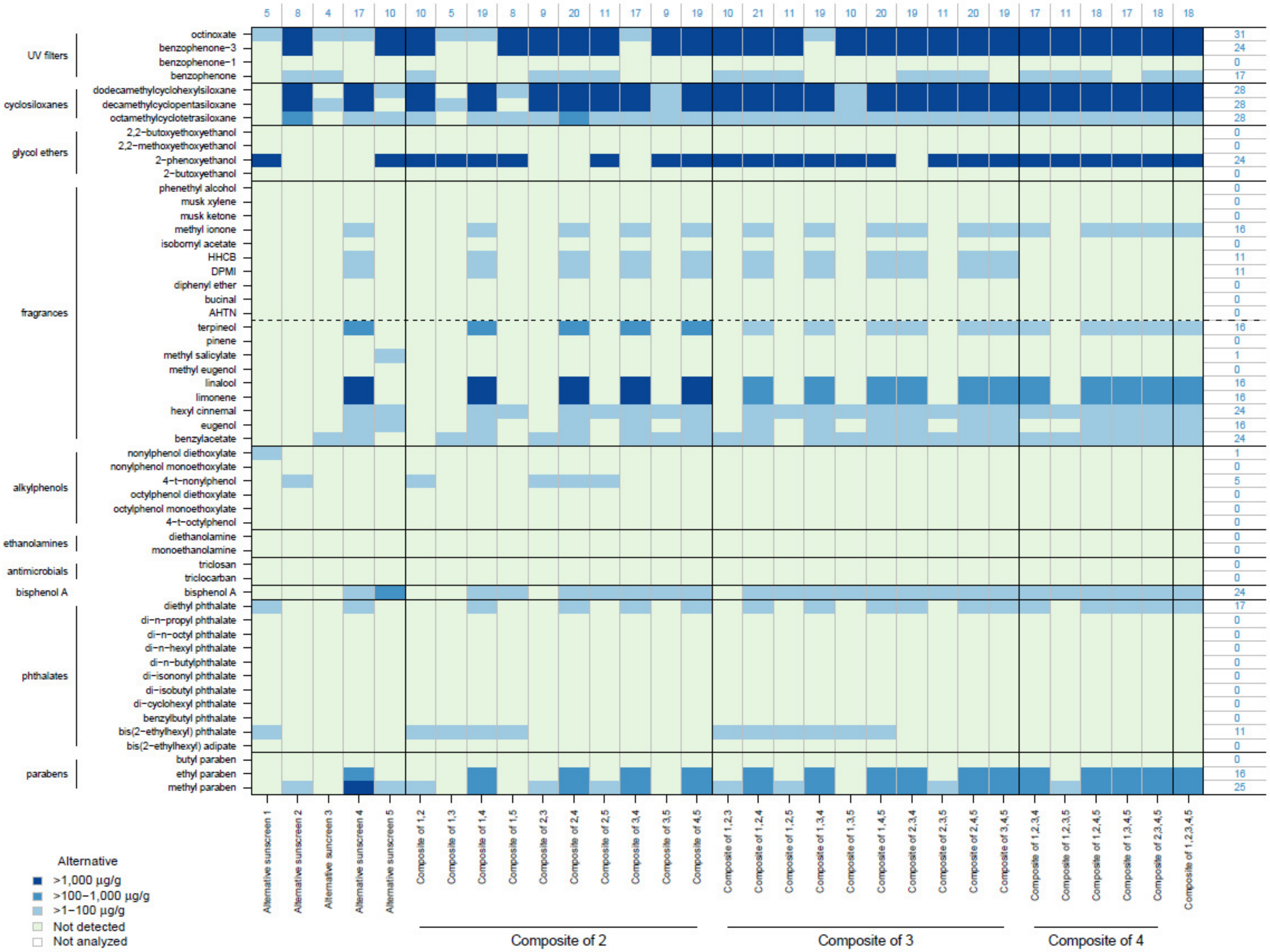

Figure S3. Effects of compositing products on detection patterns: An example using 5 alternative sunscreens. Compounds are grouped by chemical class, with dashed line distinguishing natural and synthetic fragrances. The first 5 samples are the individual sunscreen samples; the remaining are theoretical "composites" using the individual samples. Numbers in the top margin count the number of chemicals detected in composite; numbers in the right margin count number of composites containing each compound.

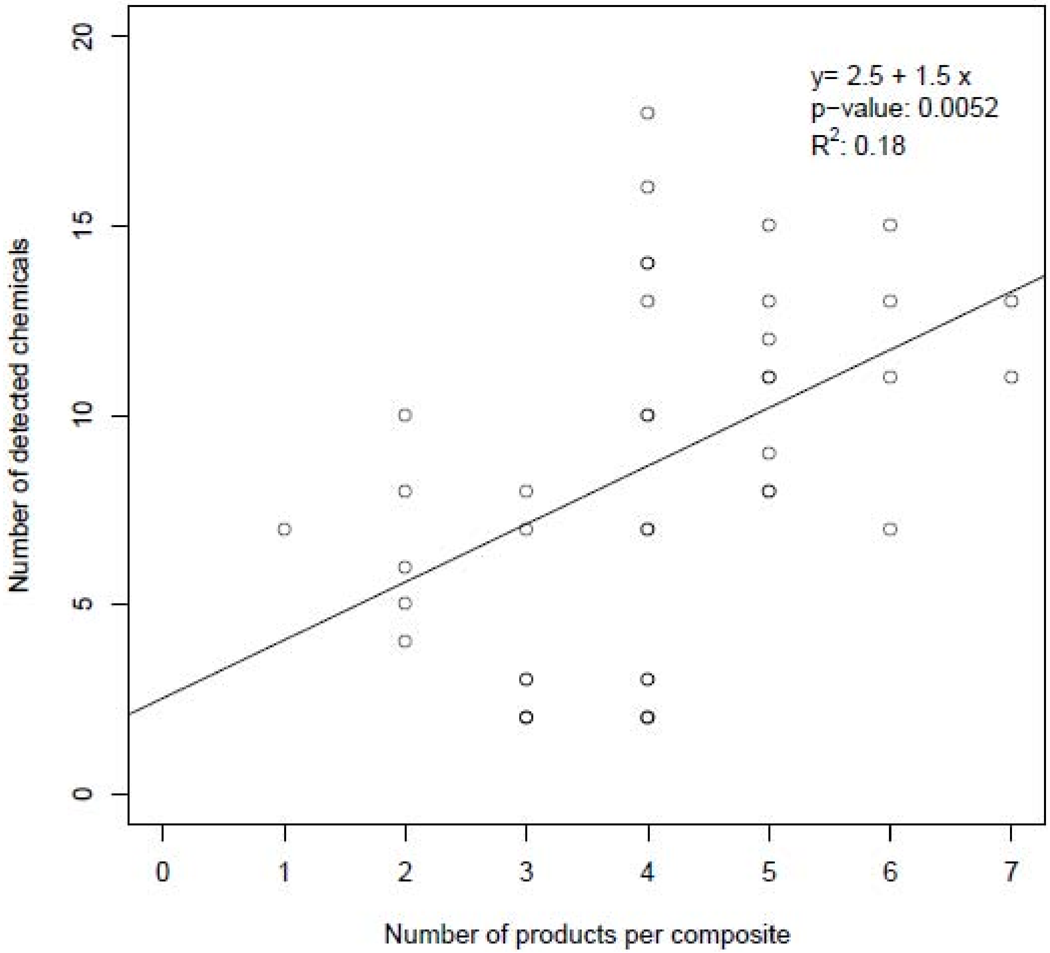

Figure S4. Number of products per composite versus number of detected chemicals for conventional samples. Linear regression model presented in upper right corner with p-value for slope estimate and R^2 value for the model. The number of products per composite and number of chemicals detected have a significant positive relationship.

Analytical Quality Assurance/Quality Control (QA/QC) Methods and Results
To composite samples: for thin liquids, a 1 ml aliquot of each sample was combined and mixed, then 0.25 mL was removed and spiked with the surrogate recovery standards (SRSs), diluted with 50 mL of 3:1 dichloromethane (DCM):methanol, ultra-sonified for 1 min and placed on a shaker table for 15 min; for thick liquids (e.g., toothpaste) and semi-solid materials (e.g., lipstick), approximately 1 g of each product was added in sequence to a tared vial and then mixed with a spatula; 0.25 g was removed, spiked with the SRSs, mixed with 0.5 g muffled Extrelute to form a free-flowing mixture, ultra-sonified and shaken in 50 mL 3:1 DCM:methanol, and filtered as needed through a syringe filter; for solids, equal amounts of each product were finely divided, mixed and 0.25 g was removed, spiked with the SRSs, and extracted as described above. A 6 mL aliquot of each extract was passed through a weak anion exchange SPE cartridge (DSC-NH2, 100 mg; Supelco), 1 mL was removed, spiked with the neutral internal standard (IS; bromobiphenyl), and analyzed using GC/MS in the full scan mode for neutral compounds. A separate aliquot of the extract was solvent exchanged into the same volume of methyl-t-butyl ether, 1 mL was removed, spiked with the IS dibromophenol-d3 and 50 uL pyridine, derivatized with BSTFA with 1% TMCS, and then analyzed using GC/MS in the full scan mode for phenolic compounds. The same phenolic extract was reanalyzed in the multiple ionization detection mode for the multi-component nonylphenol mono- and diethoxylates. The internal standard method of quantification was based on 6 point calibration curves that spanned the range of 0.15-25 μg/mL.

QA/QC measures were conducted to ensure accuracy and reliability of measurements. To evaluate potential contamination, we analyzed eight solvent method blanks in round one and five solvent method blanks in round two. To estimate precision we analyzed eight samples in round one and five samples in round 2 as duplicates. Surrogate recoveries were used to characterize accuracy and extraction efficiency.

For each compound, the method reporting limit (MRL) was defined as the maximum of the analytical detection limit and the 90th percentile of the blank concentrations within each analytical round. The nominal analytical detection limit was 1 ug/g, and so the MRL was above 1 ug/g only in cases with detectable concentrations in the blank samples. Concentrations above the MRL were considered quantified and presented in the paper.

Potential sample contamination was evaluated using solvent method blanks (n=13). The target compounds diethyl phthalate, diethanolamine, octylphenol monoethoxylate, octylphenol diethoxylate, nonylphenol monoethoxylate and nonylphenol diethoxylate were detected in at least one blank in the first analytical round and the target compounds methyl paraben, ethyl paraben, butyl paraben, bisphenol A, triclosan, monoethanolamine, diethanolamine, octylphenol diethoxylate, 4-nonylphenol, D4 (octamethylcyclotetrasiloxane), D5 (decamethylcyclopentasiloxane), D6 (dodecamethylcyclohexylsiloxane), benzophenone-1 and benzophenone-3 were detected in at least one blank in the second analytical round. Summary statistics for the solvent blanks for each analytical round are presented in Table S2.

In order to correct potential bias in the reported values, blank correction was performed for chemicals detected in at least 75% of the blanks and were specific to each analytical round. In analytical round one, none of the compounds required blank correction. In analytical round two, one compound (D5; decamethylcyclopentasiloxane) was blank corrected by subtracting the median blank concentration from the reported values, resulting in a median 87% change and maximum 200% change to reported concentration.

Precision is presented as the relative percent difference between duplicate pair concentrations (Table S3). Summary statistics were calculated only using pairs of detected values. In general, for the 28 analytes with pairs of detected values, precision estimates were <50%, except in 2 instances (ethyl paraben and 4-nonylphenol). There were 7 analytes with "mismatched" pairs, meaning that one of the duplicate pairs was detected and one was not.

Five surrogate compounds (octyl alcohol-d17, 4-chlorophenyl phenyl ether-d5, di-n-butyl phthalate-d4, phenanthrene-d10 and bisphenol A-d16) were spiked into samples prior to extraction at 1 µg/ml level (or 200 µg/g; levels of octyl alcohol-d17 increased to 2 µg/ml in the second analytical round for increased sensitivity) to evaluate accuracy and extraction efficiency. Median percent recoveries were within the 50-150% acceptance range for all surrogates over both analytical rounds (Figure S5). There were sporadic recoveries outside of this range in the 71 Round 1 samples for bisphenol A-d16 (below 50% in 5 samples and above 150% in 6 samples), and octyl alcohol-d17 (below 50% in 23 samples and above 150% in 1 sample). Variable recoveries may be attributable to interferences from complex matrices. Reported concentrations were not adjusted or corrected.

Six products were spiked prior to extraction with the full suite of analytes at concentrations of 100 ng/ml for the alkylphenol ethoxylates and 2.5 µg/ml for all other compounds, and analyzed to assess recovery. Three types of conventional and alternative products were selected to represent different matrices: glass cleaner (liquid), sunscreen (cream), and scrubbing powder (semi-solid). Thirteen recovery estimates (of 406 possible) could not be estimated due to matrix interference. Median recoveries across all products were within 50-150%, except for benzophenone-2 (not detected in any samples), monoethanolamine, and diethanolamine, which had low recoveries (Table S4). Therefore, the results presented for these compounds may be underestimates of the true concentrations. There were no significant differences in recoveries across product type ($p>0.05$).

Table S2. Summary statistics for solvent method blanks (μg/g equivalent[a]) in each analytical round.

		Round 1						Round 2					
Compound	Chemical Class	No. of Blanks	No. of Detects	Min.	Median	Max.	MRL	No. of Blanks	No. of Detects	Min.	Median	Max.	MRL
methyl paraben	parabens	8	0	0	0	0	1	5	1	0	0	2	1.2
ethyl paraben	parabens	8	0	0	0	0	1	5	1	0	0	2	1.2
butyl paraben	parabens	8	0	0	0	0	1	5	1	0	0	4	2.4
bis(2-ethylhexyl) adipate	phthalates	8	0	0	0	0	1	5	0	0	0	0	1
bis(2-ethylhexyl) phthalate	phthalates	8	0	0	0	0	1	5	0	0	0	0	1
benzylbutyl phthalate	phthalates	8	0	0	0	0	1	5	0	0	0	0	1
di-amyl phthalate	phthalates	8	0	0	0	0	1	5	0	0	0	0	1
di-cyclohexyl phthalate	phthalates	8	0	0	0	0	1	5	0	0	0	0	1
di-isobutyl phthalate	phthalates	8	0	0	0	0	1	5	0	0	0	0	1
di-isononyl phthalate	phthalates	8	0	0	0	0	1	5	0	0	0	0	1
di-n-butylphthalate	phthalates	8	0	0	0	0	1	5	0	0	0	0	1
di-n-hexyl phthalate	phthalates	8	0	0	0	0	1	5	0	0	0	0	1
di-n-octyl phthalate	phthalates	8	0	0	0	0	1	5	0	0	0	0	1
di-n-propyl phthalate	phthalates	8	0	0	0	0	1	5	0	0	0	0	1
diethyl phthalate	phthalates	8	2	0	0	4	2.6	5	0	0	0	0	1
bisphenol A	bisphenol A	8	0	0	0	0	1	5	1	0	0	4	2.4
1,4-dichlorobenzene	antimicrobials	8	0	0	0	0	1	5	0	0	0	0	1
o-phenylphenol	antimicrobials	8	0	0	0	0	1	5	0	0	0	0	1
triclocarban	antimicrobials	8	0	0	0	0	1	5	0	0	0	0	1
triclosan	antimicrobials	8	0	0	0	0	1	5	1	0	0	4	2.4
monoethanolamine	ethanolamines	8	0	0	0	0	1	5	1	0	0	4	2.4
diethanolamine	ethanolamines	8	1	0	0	2	1	5	1	0	0	2	1.2
4-t-octylphenol	alkylphenols	8	0	0	0	0	1	5	0	0	0	0	1
octylphenol monoethoxylate	alkylphenols	8	4	0	0.022	0.15	1	5	0	0	0	0	1
octylphenol diethoxylate	alkylphenols	8	5	0	0.04	0.35	1	5	1	0	0	1	1
4-t-nonylphenol	alkylphenols	8	0	0	0	0	1	5	1	0	0	0.4	1
nonylphenol monoethoxylate	alkylphenols	8	2	0	0	0.35	1	5	0	0	0	0	1
nonylphenol diethoxylate	alkylphenols	8	3	0	0	0.71	1	5	0	0	0	0	1
benzylacetate	fragrances	8	0	0	0	0	1	5	0	0	0	0	1
eugenol	fragrances	8	0	0	0	0	1	5	0	0	0	0	1
hexyl cinnemal	fragrances	8	0	0	0	0	1	5	0	0	0	0	1
limonene	fragrances	8	0	0	0	0	1	5	0	0	0	0	1
linalool	fragrances	8	0	0	0	0	1	5	0	0	0	0	1
methyl eugenol	fragrances	8	0	0	0	0	1	5	0	0	0	0	1
methyl salicylate	fragrances	8	0	0	0	0	1	5	0	0	0	0	1

		Round 1						Round 2					
Compound	Chemical Class	No. of Blanks	No. of Detects	Min.	Median	Max.	MRL	No. of Blanks	No. of Detects	Min.	Median	Max.	MRL
pinene	fragrances	8	0	0	0	0	1	5	0	0	0	0	1
terpineol	fragrances	8	0	0	0	0	1	5	0	0	0	0	1
AHTN	fragrances	8	0	0	0	0	1	5	0	0	0	0	1
bucinal	fragrances	8	0	0	0	0	1	5	0	0	0	0	1
diphenyl ether	fragrances	8	0	0	0	0	1	5	0	0	0	0	1
DPMI	fragrances	8	0	0	0	0	1	5	0	0	0	0	1
HHCB	fragrances	8	0	0	0	0	1	5	0	0	0	0	1
isobornyl acetate	fragrances	8	0	0	0	0	1	5	0	0	0	0	1
methyl ionone	fragrances	8	0	0	0	0	1	5	0	0	0	0	1
musk ketone	fragrances	8	0	0	0	0	1	5	0	0	0	0	1
musk xylene	fragrances	8	0	0	0	0	1	5	0	0	0	0	1
phenethyl alcohol	fragrances	8	0	0	0	0	1	5	0	0	0	0	1
2-isopropoxyethanol	glycol ethers	NA	NA	NA	NA	NA	NA	5	0	0	0	0	1
2-propoxyethanol	glycol ethers	NA	NA	NA	NA	NA	NA	5	0	0	0	0	1
2-butoxyethanol	glycol ethers	8	0	0	0	0	1	5	0	0	0	0	1
2-phenoxyethanol	glycol ethers	NA	NA	NA	NA	NA	NA	5	0	0	0	0	1
2-benzyloxyethanol	glycol ethers	NA	NA	NA	NA	NA	NA	5	0	0	0	0	1
2,2-methoxyethoxyethanol	glycol ethers	8	0	0	0	0	1	5	0	0	0	0	1
2,2-ethoxyethoxyethanol	glycol ethers	NA	NA	NA	NA	NA	NA	5	0	0	0	0	1
2,2-butoxyethoxyethanol	glycol ethers	NA	NA	NA	NA	NA	NA	5	0	0	0	0	1
8:2 FTOH	perfluorinated	8	0	0	0	0	1	5	0	0	0	0	1
octamethylcyclotetrasiloxane	cyclosiloxanes	NA	NA	NA	NA	NA	NA	5	3	0	2	6	5.2
decamethylcyclopentasiloxane[b]	cyclosiloxanes	NA	NA	NA	NA	NA	NA	5	5	2	6	32	17
dodecamethylcyclohexylsiloxane	cyclosiloxanes	NA	NA	NA	NA	NA	NA	5	2	0	0	2	2
3,4-methylbenzylidene camphor	UV filters	NA	NA	NA	NA	NA	NA	5	0	0	0	0	1
benzophenone	UV filters	NA	NA	NA	NA	NA	NA	5	0	0	0	0	1
benzophenone-1	UV filters	NA	NA	NA	NA	NA	NA	5	1	0	0	2	1.2
benzophenone-2	UV filters	NA	NA	NA	NA	NA	NA	5	0	0	0	0	1
benzophenone-3	UV filters	NA	NA	NA	NA	NA	NA	5	1	0	0	8	4.8
octinoxate	UV filters	NA	NA	NA	NA	NA	NA	5	0	0	0	0	1
octyl dimethyl PABA	UV filters	NA	NA	NA	NA	NA	NA	5	0	0	0	0	1

NA indicates "not applicable" since compound was added during second analytical round
MRL represents the method reporting limit, which is the maximum of the 90th percentile of the blanks or the analytical detection limit (1 ug/g). MRLs have been blank corrected when necessary.

[a] Calculated assuming 50 ml extract volume and 0.25 g sample, which was used for all product samples

[b] Subjected to blank correction by subtracting median blank concentration from reported values

Table S3. Summary statistics for precision, calculated as relative percent difference (%).

Compound	Chemical Class	No. of Duplicate Pairs	No. of Detected Pairs	No. of Mismatched Pairs[a]	No. of Nondetect Pairs	Median[b]	Max.[b]
methyl paraben	parabens	9	3	0	6	22	38
ethyl paraben	parabens	9	1	0	8	67	67
butyl paraben	parabens	9	2	0	7	36	54
bis(2-ethylhexyl) adipate	phthalates	9	0	0	9	ND	ND
bis(2-ethylhexyl) phthalate	phthalates	9	3	1	5	46	67
benzylbutyl phthalate	phthalates	9	0	0	9	ND	ND
di-amyl phthalate	phthalates	9	0	0	9	ND	ND
di-cyclohexyl phthalate	phthalates	9	0	0	9	ND	ND
di-isobutyl phthalate	phthalates	9	0	1	8	ND	ND
di-isononyl phthalate	phthalates	9	0	0	9	ND	ND
di-n-butylphthalate	phthalates	9	0	1	8	ND	ND
di-n-hexyl phthalate	phthalates	9	0	1	8	ND	ND
di-n-octyl phthalate	phthalates	9	0	0	9	ND	ND
di-n-propyl phthalate	phthalates	9	0	0	9	ND	ND
diethyl phthalate	phthalates	9	3	1	5	0.84	6.9
bisphenol A	bisphenol A	9	1	0	8	0	0
1,4-dichlorobenzene	antimicrobials	9	0	0	9	ND	ND
o-phenylphenol	antimicrobials	9	0	0	9	ND	ND
triclocarban	antimicrobials	9	0	0	9	ND	ND
triclosan	antimicrobials	9	0	0	9	ND	ND
monoethanolamine	ethanolamines	9	1	0	8	36	36
diethanolamine	ethanolamines	9	0	0	9	ND	ND
4-t-octylphenol	alkylphenols	9	0	0	9	ND	ND
octylphenol monoethoxylate	alkylphenols	9	5	0	4	21	67
octylphenol diethoxylate	alkylphenols	9	7	0	2	45	75
4-t-nonylphenol	alkylphenols	9	2	0	7	64	100
nonylphenol monoethoxylate	alkylphenols	9	1	3	4	7.5	7.5
nonylphenol diethoxylate	alkylphenols	9	4	2	3	50	83
benzylacetate	fragrances	9	2	0	7	12	18
eugenol	fragrances	9	2	0	7	5.2	10
hexyl cinnemal	fragrances	9	2	0	7	7.8	9.8
limonene	fragrances	9	3	0	6	0	7.2
linalool	fragrances	9	2	0	7	11	12
methyl eugenol	fragrances	9	1	0	8	0	0
methyl salicylate	fragrances	9	1	0	8	0	0
pinene	fragrances	9	1	0	8	8.7	8.7
terpineol	fragrances	9	0	0	9	ND	ND

Compound	Chemical Class	No. of Duplicate Pairs	No. of Detected Pairs	No. of Mismatched Pairs[a]	No. of Nondetect Pairs	Median[b]	Max.[b]
AHTN	fragrances	9	2	0	7	16	18
bucinal	fragrances	9	2	0	7	9.7	11
diphenyl ether	fragrances	9	0	0	9	ND	ND
DPMI	fragrances	9	0	0	9	ND	ND
HHCB	fragrances	9	2	0	7	12	21
isobornyl acetate	fragrances	9	0	0	9	ND	ND
methyl ionone	fragrances	9	2	0	7	12	15
musk ketone	fragrances	9	1	0	8	3.5	3.5
musk xylene	fragrances	9	0	0	9	ND	ND
phenethyl alcohol	fragrances	9	1	0	8	13	13
2-isopropoxyethanol	glycol ethers	1	0	0	1	ND	ND
2-propoxyethanol	glycol ethers	1	0	0	1	ND	ND
2-butoxyethanol	glycol ethers	9	0	0	9	ND	ND
2-phenoxyethanol	glycol ethers	1	1	0	0	6.6	6.6
2-benzyloxyethanol	glycol ethers	1	0	0	1	ND	ND
2,2-methoxyethoxyethanol	glycol ethers	9	0	0	9	ND	ND
2,2-ethoxyethoxyethanol	glycol ethers	1	0	0	1	ND	ND
2,2-butoxyethoxyethanol	glycol ethers	1	0	0	1	ND	ND
8:2 FTOH	perfluorinated	9	0	0	9	ND	ND
octamethylcyclotetrasiloxane	cyclosiloxanes	1	0	0	1	ND	ND
decamethylcyclopentasiloxane	cyclosiloxanes	1	0	0	1	ND	ND
dodecamethylcyclohexylsiloxane	cyclosiloxanes	1	0	0	1	ND	ND
3,4-methylbenzylidene camphor	UV filters	1	0	0	1	ND	ND
benzophenone	UV filters	1	0	0	1	ND	ND
benzophenone-1	UV filters	1	0	0	1	ND	ND
benzophenone-2	UV filters	1	0	0	1	ND	ND
benzophenone-3	UV filters	1	0	0	1	ND	ND
octinoxate	UV filters	1	1	0	0	0	0
octyl dimethyl PABA	UV filters	1	0	0	1	ND	ND

ND indicates insufficient number of detected pairs to calculate precision

[a] Mismatched pairs are those pairs where one sample is detected and the other is not

[b] Summary statistics calculated using duplicate pairs where both samples are detected

Table S4. Summary statistics for recoveries (%) from spiked products

Compound	Chemical Class	No. of Spiked Samples	Min.	Median	Mean	Max.
methyl paraben	parabens	5	65	78	76	83
ethyl paraben	parabens	6	73	76	77	82
butyl paraben	parabens	6	77	78	79	84
bis(2-ethylhexyl) adipate	phthalates	6	96	114	112	126
bis(2-ethylhexyl) phthalate	phthalates	6	105	115	115	123
benzylbutyl phthalate	phthalates	6	92	109	111	125
di-amyl phthalate	phthalates	6	108	113	115	124
di-cyclohexyl phthalate	phthalates	6	105	116	116	125
di-isobutyl phthalate	phthalates	6	112	118	119	128
di-isononyl phthalate	phthalates	6	98	114	112	120
di-n-butyl phthalate	phthalates	6	112	116	118	126
di-n-hexyl phthalate	phthalates	6	102	112	114	126
di-n-octyl phthalate	phthalates	6	82	112	109	122
d-n-propyl phthalate	phthalates	6	112	116	117	124
diethyl phthalate	phthalates	6	110	116	116	122
bisphenol A	bisphenol A	6	74	80	81	88
1,4-dichlorobenzene	antimicrobials	6	59	94	90	113
o-phenylphenol	antimicrobials	6	78	84	84	92
triclocarban	antimicrobials	6	49.5	72	71.5	91.5
triclosan	antimicrobials	6	72	76	77	84
monoethanolamine	ethanolamines	5	0	9	14	44
diethanolamine	ethanolamines	6	0	22	33	99
4-t-octylphenol	alkylphenols	6	81	84	86	93
octylphenol monoethoxylate	alkylphenols	6	82	84	88	110
octylphenol diethoxylate	alkylphenols	6	55	62	64	76
4-t-nonylphenol	alkylphenols	5	50	67	65	78
nonylphenol monoethoxylate	alkylphenols	4	85	90	92	103
nonylphenol diethoxylate	alkylphenols	6	59	76	117	355
benzyl acetate	fragrances	6	92	100	101	110
eugenol	fragrances	6	67	73	73	78
hexyl cinnemal	fragrances	6	100	110	110	119
limonene	fragrances	6	60	90	90	118
linalool	fragrances	6	86	110	109	121
methyl eugenol	fragrances	6	101	108	108	114
methyl salicylate	fragrances	6	23	66	59	73
pinene	fragrances	6	46	80	81	118
terpineol	fragrances	6	53	96	92	113
diphenyl ether	fragrances	6	113	118	119	126
isobornyl acetate	fragrances	6	104	121	118	123
phenethyl alcohol	fragrances	6	62	92	89	102
AHTN	fragrances	6	118	123	124	131
bucinal	fragrances	6	28	70	61	80
DPMI	fragrances	6	111	122	120	124
HHCB	fragrances	6	113	118	118	122
methyl ionone	fragrances	6	114	123	121	125
musk ketone	fragrances	6	96	108	107	116
musk xylene	fragrances	6	87	115	113	132
2-isopropoxyethanol	glycol ethers	6	49	70	74	104
2-propoxyethanol	glycol ethers	6	34	59	58	74

Compound	Chemical Class	No. of Spiked Samples	Min.	Median	Mean	Max.
2-butoxyethanol	glycol ethers	5	52	91	88	114
2-phenoxyethanol	glycol ethers	5	78	82	86	99
2-benzyloxyethanol	glycol ethers	6	5	78	67	83
2,2-methoxyethoxyethanol	glycol ethers	6	46	101	92	117
2,2-ethoxyethoxyethanol	glycol ethers	6	97	105	104	107
2,2-butoxyethoxyethanol	glycol ethers	6	50	84	77	96
8:2 FTOH	perflorinated	6	94	116	115	143
octamethylcyclotetrasiloxane	cyclosiloxanes	6	51	90	91	133
decamethylcyclopentasiloxane	cyclosiloxanes	5	81	105	108	135
dodecamethylcyclohexylsiloxane	cyclosiloxanes	5	105	119	118	130
3,4-methylbenzylidene camphor	UV filters	6	116	119	120	126
benzophenone	UV filters	6	104	116	115	122
benzophenone-1	UV filters	6	37	70	64	80
benzophenone-2	UV filters	6	0	18	17	34
benzophenone-3	UV filters	4	33	64	58	70
octinoxate	UV filters	4	130	161	154	165
octadimethyl PABA	UV filters	6	92	112	110	118

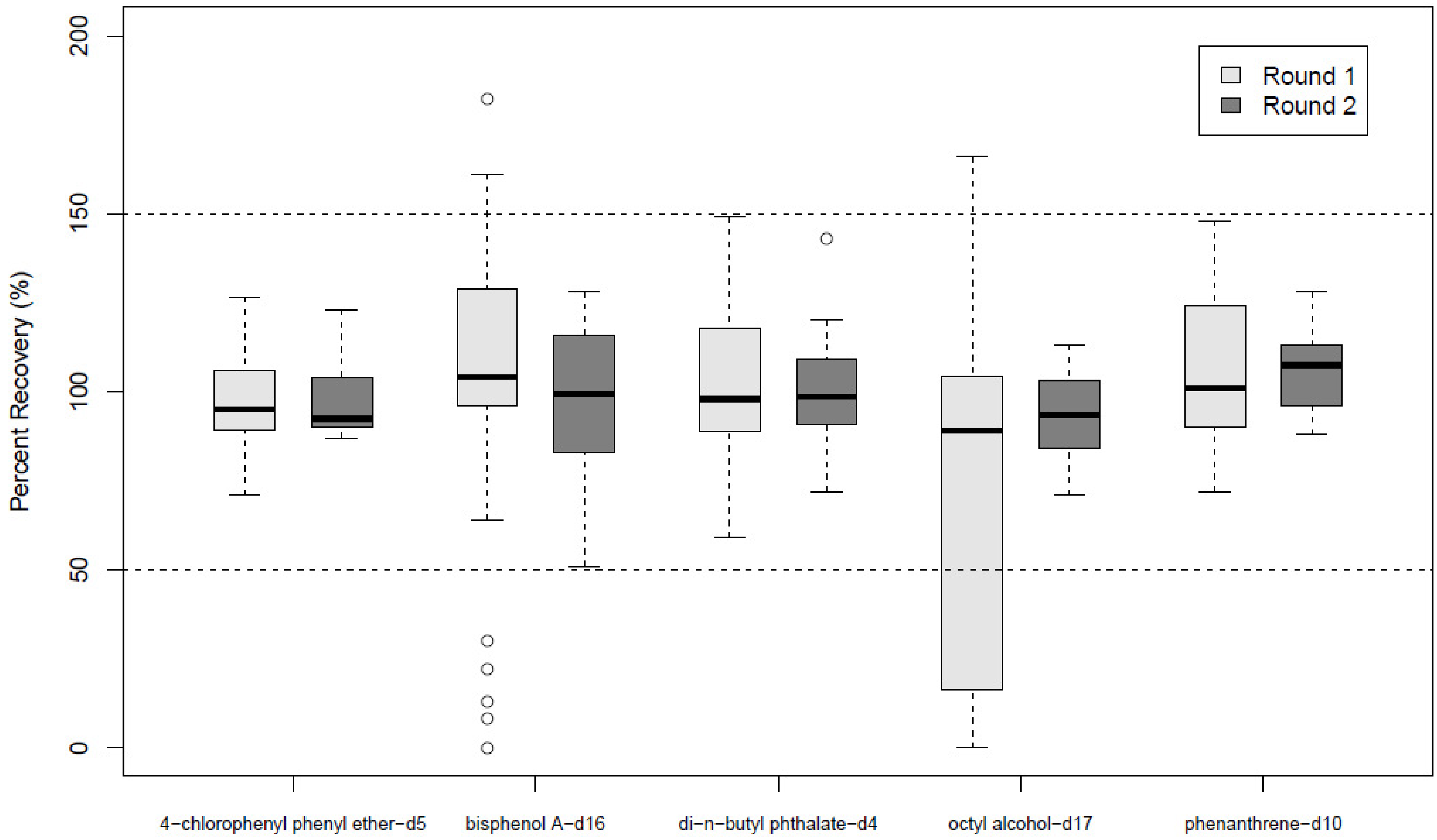

Figure S5. Surrogate recovery distributions for five surrogates for each analytical round. For each surrogate standard, there are 71 samples from Round 1 and 14 samples from Round 2. Surrogates were spiked at 1 µg/ml (200 µg/g), except for octyl alcohol-d17 in Round 2, which as increased to 2 µg/ml (400 µg/g) to increase sensitivity.

References

Bitsch N, Dudas C, Korner W, Failing K, Biselli S, Rimkus G, et al. 2002. Estrogenic activity of musk fragrances detected by the E-Screen assay using human MCF-7 cells. Arch Environ Contam Toxicol 43:257-264.

Bornehag CG, Sundell J, Weschler CJ, Sigsgaard T, Lundgren B, Hasselgren M, et al. 2004. The association between asthma and allergic symptoms in children and phthalates in house dust: A nested case-control study. Environ Health Perspect 112(14):1393-1397.

Bornehag CG, Nanberg E. 2010. Phthalate exposure and asthma in children. Int J Androl 33:333-345.

Chen JG, Ahn KC, Gee NA, Ahmed MI, Duleba AJ, Zhao L, et al. 2008. Triclocarban enhances testosterone action: A new type of endocrine disruptor? Endocrinology 149(3):1173-1179.

Choi H, Schmidbauer N, Sundell J, Hasselgren M, Spengler JD, Bornehag CG. 2010. Common household chemicals and the allergy risks in pre-school age children. PLoS ONE 5(10):e13423.

FAO/WHO. 2010. Joint FAO/WHO Expert Meeting to Review Toxicological and Health Aspects of Bisphenol A: Summary Report including Report of Stakeholder Meeting on Bisphenol A. Food and Agriculture Organization of the United Nations and World Health Organization.

Hannas BR, Furr J, Lambright CS, Wilson VS, Foster PM, Gray LE, Jr. 2011. Dipentyl phthalate dosing during sexual differentiation disrupts fetal testis function and postnatal development of the male Sprague-Dawley rat with greater relative potency than other phthalates. Toxicol Sci 120(1):184-193.

Hauser R, Meeker JD, Duty S, Silva MJ, Calafat AM. 2006. Altered semen quality in relation to urinary concentrations of phthalate monoester and oxidative metabolites. Epidemiology 17(6):682-691.

Heindel JJ, Gulati DK, Mounce RC, Russell SR, Lamb JCt. 1989. Reproductive toxicity of three phthalic acid esters in a continuous breeding protocol. Fundam Appl Toxicol 12(3):508-518.

Howdeshell KL, Wilson VS, Furr J, Lambright CR, Rider CV, Blystone CR, et al. 2008. A mixture of five phthalate esters inhibits fetal testicular testosterone production in the sprague-dawley rat in a cumulative, dose-additive manner. Toxicol Sci 105(1):153-165.

Jie X, Yang W, Jie Y, Hashim JH, Liu XY, Fan QY, et al. 2010. Toxic effect of gestational exposure to nonylphenol on F1 male rats. Birth Defects Res B Dev Reprod Toxicol 89(5):418-428.

Kamijo Y, Hayashi I, Ide A, Yoshimura K, Soma K, Majima M. 2009. Effects of inhaled monoethanolamine on bronchoconstriction. J Appl Toxicol 29(1):15-19.

Kang KS, Che JH, Ryu DY, Kim TW, Li GX, Lee YS. 2002. Decreased sperm number and motile activity on the F1 offspring maternally exposed to butyl p-hydroxybenzoic acid (butyl paraben). J Vet Med Sci 64(3):227-235.

Kumar P, Caradonna-Graham VM, Gupta S, Cai X, Rao PN, Thompson J. 1995. Inhalation challenge effects of perfume scent strips in patients with asthma. Ann Allergy Asthma Immunol 75:429-433.

Makela R, Kauppi P, Suuronen K, Tuppurainen M, Hannu T. 2011. Occupational asthma in professional cleaning work: a clinical study. Occup Med (Lond) 61(2):121-126.

Meeker JD, Calafat AM, Hauser R. 2009. Urinary metabolites of di(2-ethylhexyl) phthalate are associated with decreased steroid hormone levels in adult men. J Androl 30(3):287-297.

Mendiola J, Meeker JD, Jorgensen N, Andersson AM, Liu F, Calafat AM, et al. In press. Urinary concentrations of di(2-ethylhexyl) phthalate metabolites and serum reproductive hormones: Pooled analysis of fertile and infertile men. J Androl.

NTP-CERHR. 2008. NTP-CERHR Monograph on the Potential Human Reproductive and Developmental Effects of Bisphenol A. NIH 08-5994. Research Triangle Park, NC:National Toxicology Program Center for the Evaluation of Risks to Human Reproduction.

Orton F, Rosivatz E, Scholze M, Kortenkamp A. 2011. Widely Used Pesticides with Previously Unknown Endocrine Activity Revealed as in Vitro Anti-Androgens. Environ Health Perspect 119:794-800.

Piipari R, Tuppurainen M, Tuomi T, Mantyla L, Henriks-Eckerman ML, Keskinen H, et al. 1998. Diethanolamine-induced occupational asthma, a case report. Clin Exp Allergy 28(3):358-362.

Quinn AL, Regan JM, Tobin JM, Marinik BJ, McMahon JM, McNett DA, et al. 2007. In vitro and in vivo evaluation of the estrogenic, androgenic, and progestagenic potential of two cyclic siloxanes. Toxicol Sci 96(1):145-153.

Savonius B, Keskinen H, Tuppurainen M, Kanerva L. 1994. Occupational asthma caused by ethanolamines. Allergy 49(10):877-881.

Schlumpf M, Schmid P, Durrer S, Conscience M, Maerkel K, Henseler M, et al. 2004. Endocrine activity and developmental toxicity of cosmetic UV filters - an update. Toxicology 205(1-2):113-122.

Schreurs RH, Sonneveld E, Jansen JH, Seinen W, van der Burg B. 2005. Interaction of polycyclic musks and UV filters with the estrogen receptor (ER), androgen receptor (AR), and progesterone receptor (PR) in reporter gene bioassays. Toxicol Sci 83(2):264-272.

Seinen W, Lemmen JG, Pieters RHH, Verbruggen EMJ, van der Burg B. 1999. AHTN and HHCB show weak estrogic - but not uterotrophic activity. Toxicol Lett 111:161-168.

Stoker TE, Gibson EK, Zorrilla LM. 2010. Triclosan exposure modulates estrogen-dependent responses in the female wistar rat. Toxicol Sci 117(1):45-53.

Swan SH, Main KM, Liu F, Stewart SL, Kruse RL, Calafat AM, et al. 2005. Decrease in anogenital distance among male infants with prenatal phthalate exposure. Environ Health Perspect 113(8):1056-1061.

van der Burg B, Schreurs R, van der Linden S, Seinen W, Brouwer A, Sonneveld E. 2008. Endocrine effects of polycyclic musks: do we smell a rat? Int J Androl 31(2):188-193.

Wang R, Moody RP, Koniecki D, Zhu J. 2009. Low molecular weight cyclic volatile methylsiloxanes in cosmetic products sold in Canada: Implications for dermal exposure. Environ Int 35:900-904.

White S, Fenton S, Hines E. 2011. Endocrine disrupting properties of perfluorooctanoic acid. J Steroid Biochem Mol Biol 127(1-2):16-26.
